# Role of Mitogen-Activated Protein (MAP) Kinase Pathways in Metabolic Diseases

**DOI:** 10.14293/genint.14.1.004

**Published:** 2024-01-17

**Authors:** Gavin Yong Quan Ng, Zachary Wai-Loon Loh, David Y. Fann, Karthik Mallilankaraman, Thiruma V. Arumugam, M. Prakash Hande

**Affiliations:** 1 Department of Physiology, Yong Loo Lin School of Medicine, National University of Singapore, Singapore; 2 Department of Pharmacology, Yong Loo Lin School of Medicine, National University of Singapore, Singapore; 3 Healthy Longevity Translational Research Programme, Yong Loo Lin School of Medicine, National University of Singapore, Singapore; 4 School of Pharmacy, Sungkyunkwan University, Suwon, Republic of Korea; 5 Department of Physiology, Anatomy & Microbiology, School of Life Sciences, La Trobe University, Bundoora, Victoria, Australia

**Keywords:** MAPKs, ERK, JNK, p38, signalling, physiology, metabolism, metabolic diseases

## Abstract

Physiological processes that govern the normal functioning of mammalian cells are regulated by a myriad of signalling pathways. Mammalian mitogen-activated protein (MAP) kinases constitute one of the major signalling arms and have been broadly classified into four groups that include extracellular signal-regulated protein kinase (ERK), c-Jun N-terminal kinase (JNK), p38, and ERK5. Each signalling cascade is governed by a wide array of external and cellular stimuli, which play a critical part in mammalian cells in the regulation of various key responses, such as mitogenic growth, differentiation, stress responses, as well as inflammation. This evolutionarily conserved MAP kinase signalling arm is also important for metabolic maintenance, which is tightly coordinated via complicated mechanisms that include the intricate interaction of scaffold proteins, recognition through cognate motifs, action of phosphatases, distinct subcellular localisation, and even post-translational modifications. Aberration in the signalling pathway itself or their regulation has been implicated in the disruption of metabolic homeostasis, which provides a pathophysiological foundation in the development of metabolic syndrome. Metabolic syndrome is an umbrella term that usually includes a group of closely associated metabolic diseases such as hyperglycaemia, hyperlipidaemia, and hypertension. These risk factors exacerbate the development of obesity, diabetes, atherosclerosis, cardiovascular diseases, and hepatic diseases, which have accounted for an increase in the worldwide morbidity and mortality rate. This review aims to summarise recent findings that have implicated MAP kinase signalling in the development of metabolic diseases, highlighting the potential therapeutic targets of this pathway to be investigated further for the attenuation of these diseases.

## Introduction

Mitogen-activated protein kinases (MAPKs) belong to an important signalling pathway that regulate metabolic homeostasis. The MAPK pathway is a strict sensor of extracellular and intracellular fluctuations that may affect metabolic processes at the cellular, organ, or even organism level. This signalling arm acts as a signal transducer of these changes from the cell surface into the nucleus to bring about a plethora of cellular responses that aid in metabolic adaptation, thus maintaining normal cellular physiology. Broadly, MAPKs can be categorised into typical or atypical MAPKs.

### Typical MAPKs

**Figure 1:**
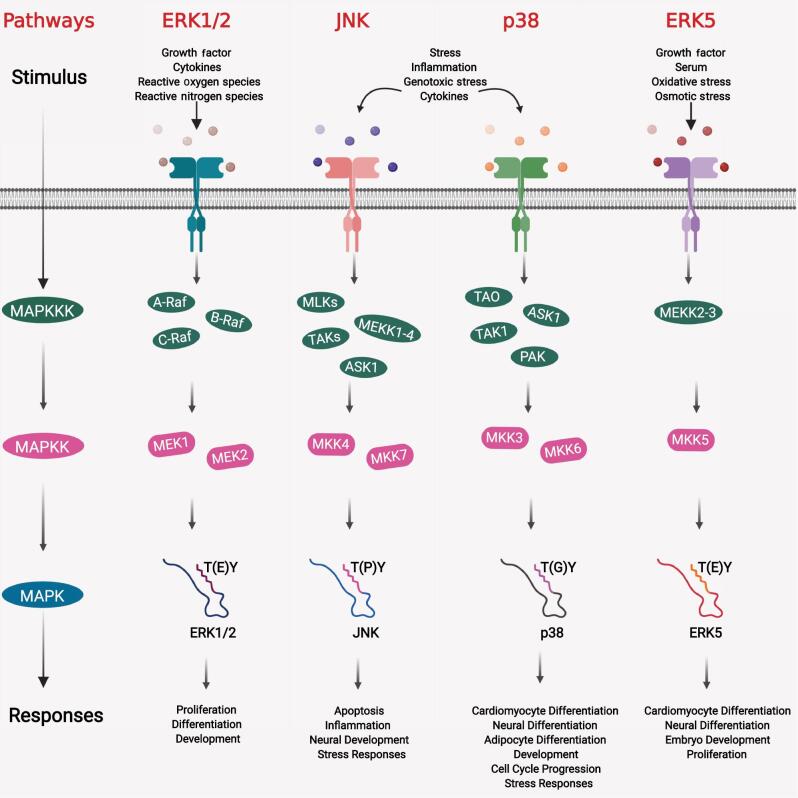
Typical MAPKs pathways. Typical MAPKs pathways consist of ERK1/2, JNK1/2/3, p38α/β/γ/δ, as well as ERK5. Each pathway responds to several types of stimuli, albeit some are overlapping. The signal transduction pathways in typical MAPKs involve three organised sequential steps. A MAPKKK will be activated in response to a stimulus, which in turn will phosphorylate a downstream MAPKK. Following which, MAPKK will be activated and phosphorylated downstream MAPKs. The members of the MAPKKK, MAPKK and MAPKs are different in each pathway. Activated MAPKK will phosphorylate a conserved tripeptide motif present in the kinase domain of the MAPKs, which is necessary to activate MAPKs. This conserved tripeptide motif consists of Thr-(X)-Tyr, where X is an amino acid that defines the different typical MAPKs as shown. For simplicity, only a partial structure of the MAPKs with its respective kinase domains (indicated via various colours) are shown. Each MAPKs pathway, in response to differential stimuli, will in turn regulate a plethora of cellular responses. The figure was created using a BioRender online software.

In mammalian cells, three typical MAPKs families have been identified, namely the conventional extracellular signal-regulated protein kinase (ERK1/2), the c-Jun N-terminal kinase (JNK1/2/3), as well as the p38 (α, β, γ, δ) isoforms signalling cascades.^[1]^ Typical MAPKs are recognised by three signalling cascade levels, where a stimulus is able to trigger the activation of a Ser/Thr MAPK kinase kinase (MAPKKK) via phosphorylation, which in turn phosphorylates and activates MAPK kinases (MAPKKs). In the last step of this three-tier cascade, MAPKK activation results in distinct dual phosphorylation of a cognate tripeptide motif (Thr-X-Tyr) found within the MAP kinase domain activation loop. Phosphorylation of the Thr and Tyr results in a conformational change in the activation loop, allowing the kinase active site to interact and activate MAPK. The amino acid (X) that is sandwiched between the conserved Thr and Tyr residues differs between the three typical MAPKs^[2]^ (Figure 1). Previously, the major focus had been directed towards the three typical MAPKs. However, another MAPK, ERK5, has been classified and is increasingly being studied. ERK5 contains the same tripeptide motif as ERK1/2. However, ERK5 differs from ERK1/2 by the possession of another transactivation domain that contains a nucleus localisation sequence at the carboxyl terminal, which is absent in the other three MAPKs families.^[3]^

#### ERK1/2 pathway

ERK1/2 is perhaps the most extensively studied MAPK pathway that mediates cellular proliferation, differentiation, and development through signalling cascades activated preferentially by interactions of various growth factors, cytokines, reactive oxygen, and nitrogen species^[4–6]^ on upstream receptors such as receptor tyrosine kinases (RTK),^[7]^ G-protein coupled receptors (GPCRs),^[8]^ as well as tumour necrosis factor receptors (TNFRs)^[9]^ (Figure 1). Receptor dimerisation and autophosphorylation of conserved tyrosine residues occur thereafter, serving as docking sites for adaptor proteins that contain the conserved Src Homology 2 (SH2) domain. The recruitment of Son of Sevenless (SOS) follows, which acts as the guanine nucleotide exchange factor (GEF) that induces the conversion of RAS-GDP to RAS-GTP. Activated RAS-GTP then triggers the recruitment and activation of RAF,^[10]^ and all RAF isoforms: namely A-RAF, B-RAF and C-RAF, are capable of activating MEK1/2, which in turn activates ERK1/2 downstream achieved by phosphorylation of their respective kinase domains.^[11–13]^ Other typical MAPK families typically signal through similar mechanisms, only differing in their interacting partners involved (Figure 1).

The outcome of activated ERK functions by phosphorylating a wide diversity of substrates ranging from protein synthesis,^[1]^ cellular proliferation and motility as well as promoting cellular migration,^[7]^ to genomic stability by increasing DNA repair protein expression.^[14,15]^ Activated ERK is able to exert pro-survival effects by overexpressing anti-apoptotic factors such as c-FLIP and Bcl-X_L_ whilst decreasing the expression of pro-apoptotic factors such as Bim and BAD.^[16]^ Although the ERK signalling arm has been conversely reported in its implication together with the nuclear factor-κB (NF-κB) pathway induced brain injury via cell death,^[17]^ neurodegeneration as well as excitotoxicity,^[18]^ its phenomenon remains largely elusive and not completely understood.

On the contrary, ERK translocates to the nucleus upon activation and phosphorylate transcription factors such as Ets, Elk-1, SAP-1, and c-Myc to promote transcription, cell proliferation, and cell cycle progression.^[1]^ Also, ERK can induce the transcriptional activation of immediate early genes such as c-Fos, which play a significant role in cellular proliferation, differentiation, and survival.^[19]^ Lastly, through the action of Msk1, ERK is also found to be able to induce chromatin remodelling and this mechanism has been reported to be critical in affecting locomotion sensitivity to drug abuse such as cocaine.^[20]^ The molecular basis of ERK behind drug abuse development is something that cannot be easily discounted.

Besides having a critical role in mitogenic growth and development, ERK1/2 plays an integral role in other physiological processes *in vivo* (Table 1). ERK1 is more ubiquitously expressed across brain structures, whereas ERK2 shows higher levels of expression in the frontal brain. Besides being highly expressed in the brain, ERK1 is also highly expressed in the intestine and placenta, whereas ERK2 is highly expressed in the heart, thymus, and skeletal muscle.^[1]^ ERK1 has been identified to be important to the development and maintenance of the immune system. Despite ERK1 knockout mice remaining viable and fertile, they displayed defective development of thymocytes, resulting in an overall reduction in CD4^+^ and CD8^+^ T cells.^[21]^ ERK1-deficient mice also display increased susceptibility to autoimmune encephalomyelitis.^[22]^ In addition, ERK1 seems to play an important role in facilitating learning and long-term potentiation,^[23]^ brain structure development,^[24]^ as well as being involved in the behavioural regulation of drug addiction.^[20]^ In addition, ERK1 knockout mice display reduced adiposity and are protected against obesity and insulin resistance,^[55]^ suggesting a link of ERK1 with metabolic maintenance. On the contrary, ERK2 knockout mice experience embryonic lethality and display defective trophoblast^[26]^ and placental development,^[27]^ hence suggesting an important role of ERK2 in embryogenesis and reproductive processes. Additionally, both ERK1/2 has been reported to be important in dentate gyrus development as well as coordinating striatal motor functions.^[24]^ Like ERK1, ERK2 is also involved in long-term memory formation and also helps to regulate social behaviours.^[28]^

**Table 1:** Characteristics and roles of typical MAPKs.

MAPKs	Tissue expression	Knockout mice phenotype	Viability of knockout mice	Possible physiological *in vivo* roles	References
ERK1	Brain Placenta Intestine	Defective differentiation of thymocytes, reduction in CD4^+^ and CD8^+^ T cells Improved striatum associated learning and memory Cocaine-induced early response gene expression and behavioural change Reduced adipogenesis, protection from obesity and insulin resistance Increased susceptibility to experimental autoimmune encephalomyelitis Lack of development in brain ventricular zones and corpus callosum with intracerebral haemorrhages	Viable, fertile (embryonic lethality if double knockout with ERK2)	Immune system maintenance Roles in autoimmune disorders Learning and memory Regulation of drug-of-abuse behavioural responses Roles in obesity Roles in diabetes Brain development	^[20–25]^
ERK2	Brain (forebrain regions) Heart Skeletal muscle Thymus	Defective trophoblast development Deficit in long-term memory formation Microcephaly, impaired cognition, and development Lack of mesoderm formation Unconventional social behaviours such as symptoms of autism-spectrum disorders, increased aggressiveness, lack of maternal responsibilities, reduced social interactions, and decreased anxiety Lack of development in brain ventricular zones and corpus callosum with intracerebral haemorrhages Abnormality in placental development	Embryonic lethality	Embryonic development Long-term memory formation Brain development Regulation of social behaviours Placental development	^[24,26–28]^
JNK1	Ubiquitous expression	Protection against diet-induced obesity and insulin resistance Lack of T cells differentiation Arthritis Inability of intestinal homeostasis and develop intestinal tumours Exacerbation of dextran sulphate sodium induced colitis Cardiac hypertrophy Improved explorative behaviour	Viable, fertile. (embryonic lethality if double knockout with JNK2)	Roles in obesity Roles in diabetes Immune system maintenance Roles in arthritis Intestinal physiology maintenance Roles in colitis Cardiac development Regulation of behaviour	^[29–35]^
JNK2	Ubiquitous expression	Protection from osteoarthritis Reduction in Type 1 Diabetes and insulitis Exacerbation of dextran sulphate sodium induced colitis Inability of differentiation of T_H_1 cells Reduction in dopaminergic neurons in MPTP-induced Parkinson disease model Absence of stress-induced impairment of contextual fear Impairment of long-term potentiation	Viable, fertile	Roles in osteoarthritis Roles in diabetes Roles in colitis Immune system maintenance Roles in Parkinson diseases Stress-induced learning of fear Long term learning	^[29,33,36–39]^
JNK3	Brain (high expression) Testis (low expression) Heart (low expression)	Absence of apoptosis in hippocampus neurons following excitotoxicity Reduction in dopaminergic neurons in MPTP-induced Parkinson disease model Absence of stress-induced impairment of contextual fear Improvement in spinal muscular atrophy Exacerbation of glaucoma due to absence of neuroprotection	Viable, fertile	Brain excitotoxicity Roles in Parkinson diseases Stress-induced learning of fear Roles in spinal muscular atrophy Roles in glaucoma formation	^[38–42]^
p38α	Ubiquitous expression	Severe defective placental angiogenesis and development Lack of proper myelination in central nervous system Development of colon tumours Impairment in erythropoiesis	Embryonic lethality	Embryonic development Placental development Central nervous system development Roles in colon homeostasis Erythropoiesis	^[43–46]^
p38β	Ubiquitous expression	No major defects reported.	Viable and fertile		^[47,48]^
p38γ	Skeletal muscle	Attenuation of colitis and colon tumorigenesis Increased endurance exercise-induced metabolic homeostasis	Viable and fertile	Roles in colitis Roles in colon homeostasis. Regulation of metabolism influenced by exercise stress	^[46,49]^
p38δ	Testis Kidney Pancreas Small intestine Lung	Reduction in skin tumorigenesis	Viable and fertile	Roles in skin cells maintenance	^[50]^
ERK5	Ubiquitous expression Spleen (high expression) Thymus (high expression) Brain (high expression)	Impairment in angiogenesis Defective cardiac development Impairment in erythroid development Impairment in neurogenesis in olfactory bulb and cause anomalous olfactory behaviours Defective placental development. Increased adiposity	Embryonic lethality	Embryonic development Vascular development Cardiac development Erythropoiesis Roles in brain structure development Roles in placental development Roles in obesity	^[51–54]^

#### JNK pathway

The JNK pathway (consisting of JNK1/2/3), also known as stress activated protein kinase (SAPK), is strongly activated by various stress-related and genotoxic stimuli to regulate a myriad of cellular responses, such as apoptosis, inflammation, and neural development^[1]^ (Figure 1 **)**. JNK1/2 are ubiquitously expressed, whereas JNK3 is confined to tissues in neurons, testis, and cardiomyocytes.^[56]^ JNKs are activated via the action of upstream receptors such as RTKs,^[57]^ GPCRs,^[58]^ and cytokine receptors^[59]^ similar to ERK. Besides Ras, the JNK pathway can be activated by Rho GTPases, such as Cdc42 and Rac. Downstream JNK activation involves the distinct three tier phosphorylation cascade as observed in ERK1/2, and occurs within the conserved tripeptide motif (Thr–Pro–Tyr) located at the activation loop within the kinase domain by MAPKKs (MKK4 and MKK7)^[1]^ (Figure 1).

Activated JNK undergoes nuclear translocation and phosphorylates c-Jun at its transactivation domain N-terminal tail at two distinct amino acid residues: Ser-63 and Ser-73.^[60]^ This results in increased transcriptional activity of c-Jun in genes with Activator protein-1 (AP-1) promoter sequences, such as Jun-B, Jun-D, activating transcription factor 2 (ATF2), p53, ELK1, Sap-1a, and even c-Jun1 itself, thereby creating a positive feedback loop directly, or through upregulation of c-Fos resulting in increased AP-1 expression.

JNK pathways are also critical in many physiological *in vivo* roles as demonstrated by knockout mouse studies (Table 1),^[29–31,31,61–63]^ which portrayed differential roles in organ specificity. Moreover, JNK1 and JNK2 knockout mice displayed overlapping phenotypes, suggesting functional redundancy between both JNK isoforms. Most notably, JNK2 deficient mice exhibited learning impairments and associations with Parkinson’s disease pathology,^[31]^ with overlapping similarities in JNK3. In addition, contrary to its survival signalling axis, JNK regulated apoptosis and increased T_H_ cell proliferation and differentiation has been reported extensively by studying JNK1^−/−^ knockout mice. In another study, subjecting JNK3^−/−^ mice to excitotoxicity resulted in the absence of excitotoxicity-induced apoptosis in the hippocampus region.^[41]^ These studies reveal the critical role of JNK in regulating apoptosis through various mechanisms. C-Jun transactivation coupled with AP-1 activity due to JNK activation has been shown to be able to upregulate pro-apoptotic protein expression, such as Bim. Moreover, mitochondrial translocation of JNK, antagonises its phosphorylation of Bcl-2 and Bcl-X_L_, thus disrupting anti-apoptotic functions. Furthermore, JNK can also decrease Smac/Diablo inhibition on caspases, as well as stimulating cytochrome-c release from the mitochondria via complementing the action of Bid and Bax.^[64]^

#### p38 pathway

The p38 MAPK signalling pathway consists of four isoforms, namely α, β, γ, and δ, which have a strong response to environmental stresses and inflammatory cytokines^[65]^ (Figure 1), sharing overlapping stimuli with the JNK signalling pathway. p38α/β are ubiquitously expressed in mammalian cells, p38γ predominantly in skeletal muscle, and p38δ in the testis, kidney, pancreas and the small intestine and lung tissues.^[43]^ p38 is also able to modulate stress responses via RTKs and GPCRS,^[66]^ as well as plausible activation by Rho family of small GTPases, playing important roles in development, cell cycle progression, and differentiation of adipocytes, neurons and cardiomyocytes.^[65]^ While p38 and JNK pathways share numerous similarities, the three-tier phosphorylation cascade in their respective signalling pathway differs slightly^[1]^ (Figure 1).

Activated p38 targets both the cytosol and nucleus, acting either directly or indirectly on its substrates. p38α/β isoforms can phosphorylate transcription factors such as Sap-1, p53, ATF2, and CHOP, as well as other proteins such as Tau and Cdc25 directly. However, p38α/β is also able to induce the activation of other protein kinases such as MSK1/2 and MNK1/2 which in turn regulate transcription factor CREB and ATF1, as well as other proteins such as Hsp27, eIF4E, and HMG-14,^[1]^ eventually impacting cell cycle progression, differentiation, cytoskeleton remodelling, and metabolism.^[66]^ p38 and its ability to induce apoptosis under certain circumstances has also been established, involving various mechanisms, such as phosphorylating pro-apoptotic factor Bim.^[43]^ In addition, cell cycle inhibition through upregulating CDK inhibitors or downregulating cyclins by p38, was observed in muscle differentiation.^[65]^ Lastly, p38^−/−^ mice displayed embryonic lethality, defective erythropoiesis,^[66]^ defective placental development^[44]^ and myelination,^[45]^ as well as its possible involvement in colon and skin tumourigenesis.^[67]^ These studies reveal the integral roles of p38 pathways in many physiological processes (Table 1).

#### ERK5 pathway

ERK5, the fourth member of the typical MAPK family, is termed the bug MAP kinase 1 (BMK1) due to its distinctive longer carboxyl-terminal transactivation domain tail, accounting for twice the molecular size of other typical MAPKs (Figure 1). It is abundantly expressed specifically in the thymus, spleen and brain tissues, playing integral roles in the regulation of important physiological processes, such as cardiomyocyte and neural differentiation, embryonic development and cellular proliferation.^[1]^

ERK5 is activated by both stress-related and mitogenic stimuli, including osmotic and oxidative stress, as well as growth factors and serum. ERK5 signalling in response to these signals act through RTKs or GPCRs involving the three-step phosphorylation of MEKK2/3, MEK5 and subsequently ERK5 activation upon dual phosphorylation within the activation loop of the kinase domain, as observed in ERK1/2.^[3,68]^

In the cytosol, ERK5 can activate the RSK family of protein kinases, driving cellular proliferation and survival. In addition, it has been reported that ERK5 upregulates cyclin D1 expression, thereby promoting G_1_/S cell cycle progression. Moreover, nuclear translocation of ERK5 then acts upon numerous substrates including Sap-1a, myocyte enhancer factor 2 (MEF2), Bad, as well as immediate response genes c-Fos and c-Jun. Thus, it can be observed that ERK5 regulated responses share numerous similarities with ERK1/2.^[1,3,68]^

Physiologically at the organism level (Table 1), genetic ablation of ERK5 in mice results in embryonic lethality, defective angiogenesis and cardiovascular development.^[51]^ In adult ERK5 deleted mice, vascular and endothelial failures were observed, resulting in haemorrhages in various organs.^[69]^ ERK5 null mice also displayed defects in placental development,^[70]^ whereas ERK5 deficient Xenopus resulted in craniofacial and cortical neuron differentiation defects.^[71]^ The roles of ERK5 in cardiovascular development and maintenance, as well as developmental processes can be appreciated through these studies.

### Atypical MAPKs

While the above-mentioned four typical MAPK families seem well-characterised and display an organised three step sequential phosphorylation cascade process, other atypical MAPKs families have been reported which are poorly understood and characterised, consisting of ERK3/4, ERK7/8, as well as Nemo-like kinase (NLK).^[72]^ These atypical MAPKs are defined without known stimuli or responses, or where their upstream kinases are poorly understood. The stimuli that activate ERK3/4/7/8 is still poorly characterised and both families also lack a distinct MAPKKK for activation. In the case of ERK7/8, their MAPKKs are poorly characterised and ERK7 undergoes self-phosphorylation to induce autoactivation, not observed in typical MAPKs. Furthermore, atypical kinases do not share the same tripeptide motif (Thr–X–Tyr) within the kinase domain of typical MAPKs families. While the domain sequence is similar between ERK7/8 and ERK5, ERK7/8 falls under the category of atypical MAPKs due to the poor characterisation of its upstream and downstream targets. NLK is notably unique as it contains a tripeptide sequence of Thr–Gln–Glu and a domain of Ala–His–Glu at its amino terminal tail that is absent in all other families.^[1,72]^ Given its poor mechanistic characterisation of and physiological roles, this review will not be discussing the roles of atypical MAPKs in metabolic disorders.

## Regulation of MAPKs

**Figure 2:**
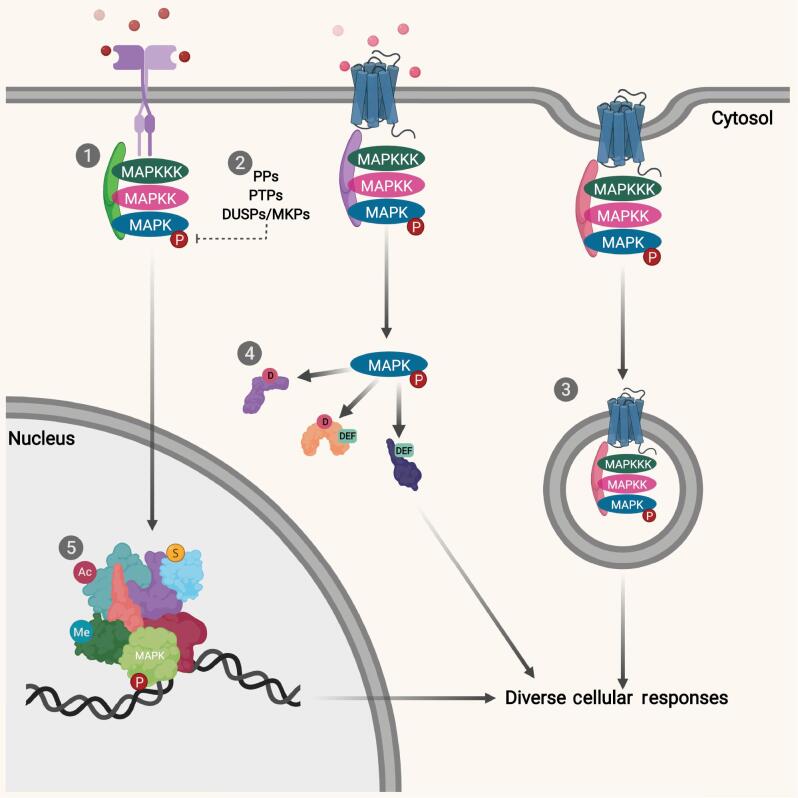
Regulations of MAPKs pathways. (1) The presence of different scaffold proteins help to sequester diverse combinations of MAPKs pathway components, which regulate the signalling of diverse combinations of downstream targets. Scaffold proteins are also important to determine the subcellular localisation of MAPKs pathway component, such as illustrated in the case of β-arrestins, where MAPKs components are shuttled towards early endosome for signalling. (2) MAPKs signalling cascades can be inactivated via the actions of phosphatases. Mono amino acid phosphatases such as PPs and PTPs catalyse the removal of phosphorylation tags from MAPKs and their components at only one amino acid sites (Ser/Thr/Tyr). On the contrary, DUSPs simultaneously remove phosphorylation tags from two amino acids positions. In MAPKs pathways, MKPs is a class of DUSPs, which remove phosphorylation from Thr and Tyr residues on activated MAPKs concurrently. (3) Localisation of MAPKs and their substrates are important not only in the regulation of the types of substrates they acted upon, but also in determining the specific cellular responses. Subcellular localisation of MAPKs and their substrates can provide a mode of spatial regulation. (4) Substrates of MAPKs signalling contain a phosphoacceptor motif, which is characterised by a distinct sequence of Ser/Thr-Pro amino acids, which is needed for the activation and function of MAPKs. Besides this cognate motif, MAPKs substrates contain specific domains called docking sites that are important in determining the specificity of interaction between activated MAPKs and their substrates. Substrates can either contain D-domain or DEF domains docking sites, which lend specificity to different types of MAPKs interactions. In some cases, substrates can also contain both D-domain and DEF domains. (5) MAPKs downstream effectors include transcription factors, which are prone to post-translational modification. The diverse combinations of post-translational modifications will bring about different transcription regulations. Overall, the tightly controlled regulatory mechanisms governing the MAPKs pathways help to regulate a myriad of cellular responses. The figure was created using a BioRender online software.

The MAPK pathways respond to diverse stimuli to bring about a diversity of cellular responses. Such complexities exhibited by the MAPK pathways are governed by intricate regulatory processes that account for the differential mechanism underlying each MAPK pathway. Many regulatory processes have been identified; namely the involvement of scaffold proteins, action of protein phosphatases (PPs), subcellular localisation of MAPKs and their substrates, the presence of cognate motifs found within substrates, as well as post-translational modifications (Figure 2).

### Scaffold proteins

Binary interactions of multi-domain scaffold proteins within MAPKs pathway components were first identified in yeast. Apart from bringing together specific MAPKKKs, MAPKKs, and MAPKs components upon stimuli challenge, scaffold proteins are able to influence the localisation of MAPKs and substrate action, segregating MAPK from other related or cross-talk pathways, as well as controlling the degree and duration of MAPK signalling to bring about differential responses.^[73]^

In the RAF/MEK/ERK pathways, various scaffolds modulate distinct ERK pathway component activity. For instance, Kinase suppressor of Ras (KSR) and β-arrestins can bind to C-Raf, MEK, and ERK for interaction. However, KSR binding restricts activated ERK to stay in the cytosol, thereby allowing ERK to act upon its cytosolic but not nuclear targets. On the contrary, β-arrestin complexes translocate to early endosomes to promote MAPK signalling. Therefore, involvement of scaffolds is important in determining the substrate types acted upon, as well as localisation differentiation. Another example, involving scaffold protein IQGAP1 participating in both EGF-mediated MAPK signalling and cytoskeleton modulation activates EGF-mediated MAPK pathway upon binding to B-Raf, Mek, and ERK. However, when IQGAP1 interacts with Cdc42 and Rac1, the actin cytoskeleton modulation pathway is activated instead. Therefore, different signalling pathways can be segregated, depending on IQGAP1 partners involved.^[73]^

In addition, scaffold proteins are also paramount in determining the kinetics and outcomes of MAPK signalling, as well as specific types of pathways activated in mediating cellular responses. Involvement of scaffold protein Grb2 after EGF challenge results only in transient activation of the pathway that drives cellular proliferation, whereas Rap1 involvement after nerve growth factor (NGF) challenge is longer-lasting.^[73]^ In addition, both growth factors can also activate the JNK pathways, via the action of JNK Interacting Protein (JIP).^[74]^

### Protein phosphatases

Activation of MAPKs requires the dual phosphorylation of their conserved Thr and Tyr residues within the cognate tripeptide motif as discussed above. As a result, inactivation of MAPKs by the removal of phosphorylation tags by phosphatases in these residues abrogate MAPKs activity. Many categories of PPs have been identified, and can be distinctly characterised into serine/threonine phosphatases (PPs), tyrosine phosphatases (PTPs), as well as dual-specificity protein phosphatases (DUSPs).^[75]^

Both PPs and PTPs are mono amino acid phosphatases, acting at only one amino acid site (Ser/Thr/Tyr). PPs such as PP2A, help to inactivate MEK1 and MEK2 at Ser positions and ERK1/2 at Thr, and together with the action of PTP, the phosphorylation tag at the Tyr position is also removed. As a result, an antagonistic effect is created by the single removal of phosphorylation in the components of the three-tier MAPK phosphorylation cascade.^[75]^

Another group of phosphatases, named DUSPs, simultaneously remove phosphorylation tags from two amino acids positions. Conventional types of DUSPs, named Mitogen-activated protein kinase phosphatases (MKPs), remove the phosphorylation from Thr and Tyr residues on activated MAPKs concurrently.^[76]^

MKPs were initially identified to have high sequence similarity to VH-1 genes found in vaccinia virus. MKPs can be further categorised into typical and atypical MKPs where each isoform in typical MPKs share similar structures. They each possess an N-terminal comprising a cluster of basic residues, a Cdc25-homology 2 (CH2) domain as well as a D-domain, which is required for the docking of activated MAPKs to their substrates, which will be discussed in detail later. At the C-terminal, MKPs contain a cluster of hydrophobic residues upstream of a catalytic domain, which possess a conserved sequence of **D**X_26_(V/L)X(V/I)H**C**XAG(I/V)S**R**SXT(I/V)XXAY(L/I)M, where “X” is defined by any amino acid. Hence, this consensus sequence at the C-terminal lends diversity to MKPs, allowing them to regulate a wide array of MAPKs activities. However, within the same consensus sequence, bolded amino acid is critical for the function of MKPs.^[77]^ There are ten isoforms of MKPs, which are found to have substrate specificities and in different cellular localisation across the cytosol and nucleus. These ten isoforms of MKPs can further be classified into three classes; the inducible nuclear MKPs (comprising DUSP1/2/4/5 or MKP-1, PAC-1, MKP-2, and hVH3 respectively), the cytoplasmic MKPs (comprising DUSP6/7/9 or MKP-3, MKP-X, and MKP-4 respectively, which have biased preference over ERK1/2 inactivation), as well as the last class with biased specificity towards JNK/p38 (comprising DUSP8/10/16 or hVH5, MKP-5,and MKP-7 respectively).^[78]^ On the contrary, although atypical MKPs are identified to contain the highest sequence homology to the VH1 gene in vaccinia virus, their domain structures differ vastly from typical MKPs by lacking the CH2 domain present in the amino tails.^[76]^ As there is a paucity in knowledge of the mechanism of action of atypical MKPs, this class of MKPs shall not be the focus in this review.

MKPs themselves are also tightly regulated via numerous mechanisms. Upon activation of MAPKs on downstream gene transcription, MKPs can be encoded as part of an immediate response gene product to deactivate MAPKs, thereby providing a negative feedback loop. Catalytically, MKPs can also serve as a form of regulation. As functional MKPs require a critical cysteine residue within its catalytic domain, redox modulation is able to influence the state of this residue, for instance, oxidation inactivates MKPs whereas reduction activates it.^[79]^ Lastly, activated MKPs can also undergo post-translational modification that will determine its kinetics and cellular localisation. For example, it has been reported that phosphorylation of activated MKP-3 induces ubiquitination of MKP-3, thereby decreasing MKP-3 half-life and also reducing its activity within cells.^[7]^ On the contrary, modifications such as the addition of a myristoyl group to activated MKPs are able to induce the transport of MKPs to the membrane, affecting subcellular localisation of MKPs.^[80]^ Overall, MKPs are particularly important in acting as molecular switches to turn off activated MAPKs activity. MKPs play various important roles in many physiological processes, and aberration of MKPs has led to the development of many diseases. MKPs are also tightly regulated as regards their function, which further adds complexity to the understanding of MKPs-mediated control over MAPKs.

### Subcellular localisation of MAPKs and their substrates

Localisation of MAPKs and their substrates are important not only in substrate regulation, but also in determining specific cellular responses. MAPKs have been reported to be concentrated at certain subcellular locations, regulating their interactions with their substrates. For example, activated ERK has been found to be shuttled to early endosomes via the action of β-arrestins, whereas KSR mediates the assembly of ERK components near the membrane, resulting in cellular component regulation.^[74]^ As mentioned, subcellular localisation of activated MAPKs also serves as potential regulators as they can only act upon substrates in the same localisation where they are concentrated. As discussed previously, activated MAPKs can act upon substrates in the cytosol or the nucleus. Precise signalling and interaction results in these MAPKs activating their substrates in their respective locations, resulting in differential durations and responses.^[74,76]^ Hence the subcellular localisation of MAPKs and their substrates may provide a mode of spatial regulation.

### Cognate motifs within substrates

Activated MAPKs can act upon numerous substrates either in the cytosol or the nucleus. However, activated MAPKs can display substrate specificity, which helps to regulate a plethora of distinct cellular responses. This substrate specificity is achieved via cognate motifs found within the substrates that can recognise specific activated MAPKs, facilitating their interactions and mediating downstream responses.

Substrates of MAPKs signalling contain a phosphoacceptor motif, characterised by the distinct sequence of Ser/Thr-Pro amino acids needed for MAPKs activation and function. Besides this cognate motif, MAPKs substrates contain specific domains called docking sites that are important in determining the specificity of interactions between activated MAPKs and their substrates.^[81]^ Many different types of docking sites have been identified and their discovery improves our current knowledge in explaining the complex mechanism behind MAPKs signalling.

One prominent docking site present in the substrates is the D-domain, also known as the δ domain. This domain is characterised by basic residue islands upstream of a cognate motif that is normally Leu–X–Leu or a triplet of hydrophobic residues. The variation in the amino acid at the “X” position of the motif, as well as the spacing between the first and last residue of the motif lend specificity to the substrate, in turn determining the specific interaction with a MAPK.^[82]^ For instance, Elk-1 is one of the substrates that contain a D-domain, sharing a similar docking site for JNK and ERK. Moreover, another docking site termed the DEF domain has been identified. The DEF domain contains the characteristic Phe/Tyr–X–Phe/Tyr–Pro, which is recognised by a specific ERK and p38α isoform. In addition, there exists many other motifs yet to be fully characterised^[1,81]^ Given the diverse combinations of motifs that each substrate can contain, this will generate a repertoire of substrate possibilities that are specific for different MAPKs, hence regulating the diverse cellular responses that MAPKs pathways are able to mediate.

### Post-translational modifications

Most MAPKs pathway downstream effectors include the involvement of transcription factors, which regulate gene responses after activation of the pathways. These transcription factors are susceptible to post-translational modifications that will determine their cellular fate. These modifications regulate the transcription factors’ interacting partners and complex assembly, which in turn will affect the type and duration of gene responses that are mediated. For example, Elk-1 transcription factor is a downstream effector of the ERK and p38 pathway. In the absence of stimuli, Elk-1 is sumoylated, which allows it to be associated with HDAC2, a co-repressor, and thus Elk-1-mediated response genes are switched off. When the ERK pathway is activated, activated ERK is able to induce the phosphorylation of Elk-1, abrogating the sumoylation status of Elk-1 and allows histone deacetylases (HDAC-2) to dissociate, thereby switching on Elk-1-mediated gene transcription.^[83]^ However, upon different stimuli such as stress signals, which activate the p38 pathway, activated p38 is able to induce the phosphorylation of both Elk-1 and another co-protein PIASxα.^[84]^ When both the transcription factor and co-protein are phosphorylated, Elk-1 is able to retain its sumoylation status but is not able to induce gene transcription as effective as the phosphorylation of Elk-1 exclusively. As such, Elk-1-mediated gene transcription is partially active under stressful stimuli. Besides sumoylation, acetylation status has also been found to recruit either histone acetyltransferase (HATs) or HDACs, which are commonly a co-activator and a co-repressor, respectively. Acetylation of transcription factors normally recruits HATs, which turn on gene transcription whereas recruitment of HDAC leads to the opposite results.^[82]^ Hence, acetylation serves as a useful switch for gene transcription through the recruitment of transcriptional complexes. Here, we attempt to use a few examples of post-translational modifications to illustrate the regulation of the MAPKs pathways. Given the existence of many different post-translational modification types, the complexities behind these regulations make the understanding of MAPKs signalling very profound.

## MAPKs Pathways Involvement in Metabolic Disorders

Metabolic disorders are diseases that arise due to disruptions in normal metabolic processes and have become a rising concern in recent years due to their associated high morbidity and mortality in populations. However, complexities arise when these disorders also have comorbidity aspects where one disorder may be the risk factor for the accelerated development of other metabolic disorders. As a result, the development of a cluster of metabolic disorders is particularly prevalent, resulting in a metabolic syndrome, in turn greatly increasing the risk of patients suffering from cardiovascular diseases, diabetes, hepatic diseases, atherosclerosis, and even stroke.^[85]^ While these disorders have plagued different populations and have placed great stress on both social and economic sectors of countries, the origin and development of these diseases are still not well understood. The MAPKs pathways are fundamentally important in regulating normal physiological processes and have also been identified to be an integral player in maintaining metabolic adaptation. Disruption of these pathways has been reported to be associated with the development of metabolic disorders. As a result, the molecular mechanisms of MAPKs and their regulations are important in understanding the pathogenesis of these diseases and hopefully highlights the potential therapeutic targets of the MAPKs pathway that can be quickly discovered from future investigations.^[86]^

### MAPKs in obesity and diabetes

Obesity is a worldwide epidemic characterised by excessive fat accumulation that eventually disrupts the normal functioning of health. Obesity has been identified to be an important risk factor for the development of other diseases, such as diabetes, cardiovascular diseases, as well as cancer.^[25]^ Regulation of adipose tissue formation and differentiation helps to maintain normal adiposity in the body. Aberration in this regulation has been shown to lead to hyperplasia of adipose tissues, exacerbating the development of obesity (Table 2).

**Table 2:** Roles of MAPKs in metabolic disorders.

Metabolic Disorders	Studies conducted on MAPKs pathways	Roles of MAPKs pathways in disease development	Potential targets	References
Obesity	ERK pathways			
	Treatment of MEK1 inhibitor, U0126, in 3T3-LI fibroblast resulted in decreased adipogenesis ERK1 deficient mice display reduced adipogenesis and protection from obesity ERK pathway inhibitor administration in ERK1 knockout mice shows no impairment in adipogenesis ERK5 knockout mice display increased appetite and adiposity	MEK1 and ERK1 required for adipogenesis in 3T3-LI fibroblast ERK1 is a driver of adipogenesis and development of obesity, but not ERK ERK5 may plays a protective role against adipogenesis and development of obesity.	MEK1 ERK1 ERK5	^[25,55,87,88]^
	p38 Isoforms pathways			
	Treatment of p38 inhibitor, SB203580, in 3T3-LI fibroblast resulted in decreased adipogenesis MKK6 overexpression induces adipogenesis	p38 required for adipogenesis in 3T3-LI fibroblast MKK6, upstream of p38, is a driver of adipogenesis	p38 MKK6	^[89,90]^
	JNK pathways			
	JNK1 deficient mice, but not JNK2, display reduced adipogenesis and protection from obesity	JNK1, but not JNK2, is a driver of adipogenesis and development of obesity	JNK1	^[32]^
	Regulatory components			
	MKP-1 knockout mice show higher ERK activation and display reduced adiposity and protection against obesity Reduction of MKP-1 using antisense oligonucleotide results in higher ERK activation and leads to reduced adiposity MKP-3 deficient mice demonstrate resistance to diet-induced obesity JIP1 deficient mice demonstrate resistance to diet-induced obesity	MKP-1 in ERK axis may be a driver/negative regulator of adipogenesis and development of obesity, depending on temporal regulation MKP-3 may be a driver of adipogenesis and development of obesity JIP1 may be a driver of adipogenesis and development of obesity through its interaction with JNK	MKP-1 MKP-3 JIP1	^[91–93]^
Diabetes	ERK pathways			
	ERK1 deficient mice display protection against insulin resistance Elevation in ERK in Type II diabetics patients’ adipocyte ERK1/2 signalling is reported to improve β-cell function in the pancreas via proliferation, and thereby improves insulin sensitivity	ERK1 may be a driver of insulin resistance ERK may contribute to the development of Type II diabetes ERK1/2 may be important for β-cell function in the pancreas, as well as being a contributing factor to insulin sensitivity	ERK1 ERK2	^[52,55,90,94]^
	p38 Isoforms pathways			
	Treatment of p38 inhibitor, SB203580 or SB202190, in 3T3-LI adipocytes and L6 muscle myotube cell lines resulted in decreased glucose transport into the cells. 3T3-LI adipocytes isolated from Type II diabetic patients resulted in increased p38 activation and decreased glucose transport into the cells. Elevation in p38 in Type II diabetics patients’ adipocyte.	Controversial roles of p38 in regulating glucose transport into cells. p38 may contribute to the development of Type II diabetes.	p38 (?)	^[52,90,95]^
	JNK pathways			
	JNK1 deficient mice display protection against insulin resistance JNK2 knockout mice demonstrate reduced type I diabetes symptoms and insulitis	JNK1 may be a driver of insulin resistance JNK2 may contributes towards the development of Type I diabetes and insulitis	JNK1 JNK2	^[32,36,52,62,96]^
	Elevation in JNK in Type II diabetics patients’ adipocyte JNK activation have resulted in β-cell failure and thereby reducing insulin secretion	JNK may contribute to the development of Type II diabetes JNK may be responsible for β-cell failure and provides a molecular mechanism on the lack of insulin secretion in diabetic patients		
	Regulatory components			
	JIP1 deficient mice show attenuation of insulin resistance p62 deficient mice results in insulin resistance MKP-3 deficient mice display improved insulin sensitivity MKP-5 deficient mice develop insulin resistance MKP-4 overexpression displays protective effect against insulin resistance	JIP1 may be a driver of insulin resistance p62 may play a protective role against insulin resistance MKP-3 may have a role in insulin resistance MKP-5 and MKP-4 may play a protective role against insulin resistance	JIP1 p62 MKP-3 MKP-4 MKP-5	^[92,97–100] [101,102]^
Atherosclerosis	ERK pathways			
	Activation of ERK1/2 in mouse macrophages after oxidised low-density lipoprotein treatment Treatment of ERK inhibitor PD98059 does not affect foam cell formation	Roles of ERK1/2 in foam cell formation ERK is not required for foam cell formation	ERK (?)	^[103]^
	p38 isoforms pathways			
	Activation of p38α in mouse macrophages after oxidised low-density lipoprotein treatment Treatment of p38 inhibitor SB203580 blocks foam cell formation MK2 knockout mice display resistance to atherosclerosis development Downregulation of p38α resulted in increased apoptosis of macrophages but not required for formation of plaque.	Roles of p38α in foam cell formation p38 is required for form cell formation Downstream p38 substrate, MK2, seems to be a driver of atherosclerosis development p38α seems to play a pro survival roles in macrophages but not involve in plaque formation	p38α MK2	^[103,104]^
	JNK pathways			
	Activation of JNK1/2 in mouse macrophages after oxidised low-density lipoprotein treatment Treatment of JNK inhibitor SP600125 blocks foam cell formation. JNK1/2 deficient mice develop resistance to atherosclerosis JNK2 knockout mice develop smaller atherosclerotic lesion	Roles of JNK1/2 in foam cell formation JNK is required for form cell formation JNK seems to be a driver of atherosclerosis development	JNK1 JNK2	^[103,105]^
	Regulatory components			
	MKP-1 deficiency in bone marrow transplantation model display accelerated atherosclerotic lesion after being fed a HFD MKP-1 and apoE deficient mice decreases the formation of atherosclerotic lesion	Controversial roles of MKP-1 in atherosclerotic lesion development	MKP-1 (?)	^[105,106]^
Cardiovascular diseases	ERK pathways			
	Administration MEK1/2 inhibitor U0126 or RAF inhibitor SB-386023 resulted in reduction in cardiac hypertrophy RAS/RAF/MEK/ERK activation in cultured myocytes resulted in modifications of ion modulators, resulting in defective sarcoplasmic reticulum calcium homeostasis ERK1/2 deficient mice display cardiac hypertrophy Overexpression of MEK1in mice display cardiac hypertrophy ERK5 activation has been detected in aortic smooth muscle cells in cardiac hypertrophic patients Genetic deletion of ERK5 as well as ERK5 inhibitor administration of BIX02189 display attenuation of cardiac hypertrophy Upstream MEK5 constitutive expression in transgenic mice shows increased cardiac hypertrophic responses, however upon ischemic/reperfusion injury, constitutive expressed MEK5 transgenic mice inhibit cardiac injury via the activation of ERK5	Upstream ERK pathway via the action of MEK1/2 may play a role in cardiac hypertrophy RAS/RAF/MEK/ERK pathways may contribute to the development of cardiac hypertrophy through defective ion channel and ion level maintenance ERK5 contributes to the pathogenesis of cardiac hypertrophy MEK5 may contribute to cardiac hypertrophy but may also play a protective role against cardiac injury	ERK MEK1 MEK2 ERK5 MEK5 (?)	^[1,65,107–110]^
	p38 isoforms pathways			
	Overexpression of p38α/β dominant negative mice display cardiac hypertrophy response, which is also displayed by wild type mice. Overexpression of dominant negative MKK3 and MKK6 in mice develop cardiac hypertrophy Overexpression of p38 in mice do not result in cardiac hypertrophy. Long term inhibition of p38 using RWJ67657 resulted in attenuation of cardiac remodelling Treatment of p38 inhibitor SB203580 and FR167653 also display reduced cardiac remodelling Pro-inflammatory cytokines production in myocytes via the p38 pathway for the induction of fibrosis and hypertrophy.	p38α/β does not seem to be involved in cardiac hypertrophy MKK3 and MKK6 may contribute to cardiac hypertrophy p38 may be involved in cardiac remodelling via inflammatory responses	MKK3 MKK6 p38	^[107,111–113]^
	JNK pathways			
	Overexpression of MKK4 in cardiomyocytes resulted in cardiac hypertrophy Overexpression of dominant negative mutant form of MKK4 reduced the hypertrophic responses JNK1/2/3 knockout mice display cardiac hypertrophy response, which is also displayed by wild type mice JNK1 knockout mice exhibited lower left ventricular systolic function during pressure overload before returning to basal level. Overexpression of MKK7 resulted in congestive heart failure in mice but not hypertrophy JNK1 knockout in mice heart resulted in elevated fibrosis when subjected to pressure overload Treatment of JNK inhibitor SP600125 to dilated cardiomyopathy hamster heart resulted in increased apoptosis and fibrosis Treatment of all trans retinoic acid to aortic banded rats downregulates JNK pathway, thereby prevent the development of cardiac remodelling	MKK4 may be involved in the development of cardiac hypertrophy JNK1/2/3 does not seem to be involved in cardiac hypertrophy JNK1 may participate a protective role in cardiac remodelling JNK may contribute to cardiac remodelling MKK7 plays a role in the contribution of congestive heart failure but not hypertrophy	MKK4 JNK1 (?) MKK7	^[111,114–117]^
	Regulatory components			
	Grb2 knockout mice show resistance to cardiac hypertrophy Overexpression of MKP-3 in mice display cardiac hypertrophy through reduction in ERK1/2 activation Treatment of all trans retinoic acid to aortic banded rats upregulates MKP-1/2 and downregulates JNK pathway, thereby prevent the development of cardiac remodelling MKP-1 knockout mice results in limited cardiac hypertrophic response MKP-1/2 double knockout mice display immense hypertrophic responses	Grb2 may be involved in the development of cardiac hypertrophy MKP-3 may be involved in the development of cardiac hypertrophy MKP-1/2 may play a protective role against cardiac remodelling MKP-1/2 may play a protective role against cardiac hypertrophy	Grb2 MKP-3 MKP-1 MKP-2	^[78,117–119]^
Stroke	ERK pathways			
	ERK1/2 have been found to be elevated, upregulating growth factors and induce hypothermia, but also cause inflammation and cerebral cell death Inhibition of ERK signalling pathway in neuronal cells subjected to ischemic conditions resulted in a decrease in the expression of NLRP1 and NLRP3 inflammasome, which in turn reduces the expression of inflammatory precursors and proteins such as IL-1β and IL-18	ERK1/2 may be both neuroprotective and damaging during ischemic stroke ERK signalling is involved in NLRP1 and NLRP3 inflammasome response in neuronal cells subjected to ischemic stroke	ERK1/2 (?) ERK	^[114,120]^
	JNK pathways			
	Inhibition of JNK signalling pathway in neuronal cells subjected to ischemic conditions resulted in a decrease in the expression of NLRP1 and NLRP3 inflammasome, which in turn reduces the expression of inflammatory precursors and proteins such as IL-1β and IL-18	JNK signalling is involved in NLRP1 and NLRP3 inflammasome response in neuronal cells subjected to ischemic stroke	JNK	^[120]^
	p38 isoforms pathways			
	p38 has been found to be elevated in peri-infarct area in astrocytes, contributing to astrogliosis Treatment of p38 inhibitor SB239063 reduced astrogliosis. p38 knockout mice also display attenuation of astrocytes migration and astrogliosis Inhibition of p38 signalling pathway in neuronal cells subjected to ischemic conditions resulted in a decrease in the expression of NLRP1 and NLRP3 inflammasome, which in turn reduces the expression of inflammatory precursors and proteins such as IL-1β and IL-18	p38 contribute to astrogliosis which leads to the damaging effects of ischemic stroke p38 signalling is involved in NLRP1 and NLRP3 inflammasome response in neuronal cells subjected to ischemic stroke	p38	^[120,121]^
	Regulatory components			
	Treatment of MKP-1 inhibitor, as well as genetic ablation of MKP-1, increase inflammation and apoptosis of neuronal and glial cells.	MKP-1 may be neuroprotective during stroke	MKP-1	^[122]^
Hepatic diseases	p38 isoforms pathways			
	p38α knockout mice resulted in reduced hepatomegaly. p38γ and p38δ knockout mice display hepatosteatosis resistance in HFD as well as MCD diet mice.	p38 may be involved in the development of hepatic diseases	p38	^[123,124]^
	JNK pathways			
	HFD mice develops steatosis via JNK1 activation MCD mice develops non-alcoholic steatohepatitis via JNK1 activation JNK1 knockdown mice using antisense oligonucleotide leads to decreased steatohepatitis and insulin resistance even when being fed HFD JNK1 induces liver injury in MCD mice JNK1/2 deficiency in bone marrow transplantation mice model develops liver fibrosis by inducing chronic inflammation via the action of JNK1 knockdown of JNK2 display steatohepatitis, which has similar phenotype as wild type mice Ablation of JNK2 resulted in increased liver injury through increased apoptosis	JNK1 may be involved in the development of steatosis, steatohepatitis, as well as liver injury JNK2 does not seem to be involved in the development of steatohepatitis but may play a protective role against liver injury	JNK1 JNK2	^[125–128]^

On the contrary, diabetes mellitus (DM) is characterised by a hyperglycaemic state, where high glucose levels can be observed in the blood over prolonged periods of time. There are two different types of DM; namely type I diabetes, characterised by an autoimmune disorder where pancreatic β-cells producing insulin are being attacked and destroyed by autoreactive immune cells. Absolute insulin deficiency occurs as a result leading to the disruption of glucose homeostasis. Meanwhile, type II diabetes is characterised by insulin resistance and impaired insulin secretion, often regarded as a metabolic disorder due to pancreatic β-cells dysfunction. Type II DM has many established risk factors, among which is obesity. Obesity-induced insulin resistance is a major risk factor for the development of diabetes.^[86]^ The MAPKs signalling pathway is implicated in insulin signalling by regulating the action of insulin receptors. Upon activation by insulin, the activated insulin receptor recruits either adaptor proteins, Grb2, or insulin receptor substrate (IRS), which in turn activates downstream ERK/p38/JNK pathways, thus regulating glucose homeostasis.^[10,129]^ Similarly, an aberration in MAPKs signalling pathway has been implicated in the development of DM, and complications arise when the risk of developing DM is confounded by obesity (Table 2).

#### ERKs in obesity and diabetes

Involvement of the MAPKs pathway in the regulation of adipose tissue formation and differentiation was first identified by *in vitro* studies using 3T3-LI fibroblast cells. Adipogenesis in 3T3-LI fibroblast cells involve the activation of MEK1 and ERK1 pathways. Treatment of MEK1-specific inhibitor U0126 resulted in the abrogation of ERK1 activation and impairment of adipogenesis. In addition, transcriptional expression of peroxisome proliferator-activated receptor (PPARγ), CCAAR/enhancer-binding protein (C/EBP), as well as, perilipin and adipocyte-specific fatty acid binding protein (aP2) were decreased upon treatment with U0126.^[87]^ As a result, this study illustrates that adipogenesis requires the activation of ERK for the transcriptional activation of genes important for adipogenesis including PPARγ, C/EBP, and aP2. However, it was reported that the involvement of ERK in adipogenesis has a temporal regulation, where ERK activation is required at the early stage of adipogenesis for proliferation of adipocytes, whereas ERK is subsequently switched off during adipocyte differentiation.^[25]^

In addition, *in vivo* studies were performed to investigate the associations between MAPKs and the development of obesity. Bost *et al*.^[25]^ described that ERK1 knockout mice display reduced adipogenesis, protection from obesity and insulin resistance.^[25]^ However, it is impossible to investigate the role of ERK2 on obesity as the deletion of ERK2 in mice led to embryonic lethality.^[26]^ When an ERK pathway inhibitor is administered in ERK1 knockout mice, no impairment in adipogenesis was observed, suggesting that ERK2 is not required for adipogenesis.^[130]^

Interestingly, ERK5 signalling seemed to be important in regulating the opposite functions as compared to other MAPKs. Mice deficient in ERK5 show increased food intake and as a result, demonstrate increased adiposity.^[88]^ Another study also reported implications behind type II DM and obesity due to ERK5 deficiency specifically in leptin receptor (LepR)-expressing neurons of female mice showing impaired glucose tolerance, food intake, energy expenditure, and decreased physical activity.^[131]^ Furthermore, studies on regulators of the MAPKs pathway can also help to account for the molecular mechanism behind obesity. For instance, a study on MKP-1 showed that MKP-1 knockout mice had lower ERK activation and manifested better protection against the development of obesity when fed with a chow diet as compared to wild type, and displayed reduced adiposity.^[91]^ However, inhibition of MKP-1 using antisense oligonucleotides resulted in the sustained activation of ERK1, which led to reduced adiposity, contrary to findings reported in MKP-1 knockout mice. This may be due to the temporal regulation of ERK in adipogenesis and differentiation. Overall, the roles of MKP-1 in influencing the risks of developing obesity highlights the potential of aberrant regulation of ERK leading to disease development. As such, there should be focus in including the plausible roles of regulators of the MAPKs pathway in their potential to cause diseases.

On the contrary, the molecular link between ERKs and insulin signalling has now been established. ERK1 knockout mice displayed protection against insulin resistance,^[55]^ and ERK is elevated in adipocytes of type II DM patients.^[52]^ Similarly, the ERK1 adaptor, p62, has also been implicated in insulin resistance. p62 is a negative regulator of ERK1, and the absence of p62 has been reported to result in insulin resistance, which coincides with earlier findings that activation of ERK1 promotes the development of obesity and diabetes.^[97]^ However, complexities arise again when ERK1/2 signalling is reported to improve β-cell function in the pancreas via proliferation, and thereby improves insulin sensitivity.^[94]^ Thus, ERK seems to be involved in the modulation of insulin resistance and what it contributes to the development of DM may be organ specific.

#### JNKs in obesity and diabetes

JNK1 knockout mice, but not JNK2 knockout mice, display protection against diet-induced obesity and insulin resistance.^[25,32,62]^ Furthermore, JIP1-deficient mice exhibit protection against diet-induced obesity when fed a high-fat diet (HFD). By interacting with JNK and subsequently resulting in JNK activation, JIP1 is able to modulate adipogenesis in obese mice.^[92]^ As such, the scaffold protein is also important in the regulation of adipogenesis. Taken together, this provides novel insight that JNK1 is an important regulator of adipogenesis and may serve as a potential therapeutic target to attenuate the development of obesity.

Moreover, JNK has also been shown to contribute to insulin resistance, exacerbating the development of DM. JNK is found to be elevated in adipocytes of type II diabetic patients,^[52]^ and JNK2 knockout mice have also been reported to show reduced type I diabetes symptoms and insulitis.^[36]^ Moreover, in conjunction with the findings that JNK and JIP1 deficiency resulted in protection against development of obesity, insulin sensitivity is also observed in both knockout mice models. Both JNK and JIP1 induce phosphorylation of Ser residues in adaptor IRS, which is known to contribute to insulin resistance. The absence of both components may thereby attenuate insulin resistance. JNK participation in pancreatic cell regulation of insulin secretion has also been reported. Under diabetic conditions, oxidative stress is induced, which in turn activates this stress-related response pathway. Prolonged JNK activation has been shown to lead to β-cell failure and thereby reducing insulin secretion overall.^[92]^ As a result, given the significant contributions of JNK in DM, it is therefore crucial to investigate this aspect further.

#### p38 in obesity and diabetes

To investigate the plausible roles of the p38 pathway in adipogenesis, a p38 inhibitor, SB203580, was administered to 3T3-LI fibroblast cells whereby adipogenesis was decreased via a reduction in C/EBPβ transcription. Bone morphogenic protein 2 (BMP-2) challenge coupled with p38 inhibitor administration reveals the roles of p38 in regulating adipogenesis via the action of PPARγ transcriptional activation.^[132]^ Lastly, overexpression of MKK6 also induces adipogenesis.^[89]^ Taken together, these studies describe the positive roles of p38 in adipogenesis. Furthermore, p38 has been implicated in the development of diabetes. As p38 knockout mice display embryonic lethality, *in vitro* studies using L6 muscle myotube cell lines and 3T3-L1 adipocytes demonstrate the roles of p38 in regulating glucose transport, as inhibitor studies using p38 inhibitor SB202190 or SB203580 resulted in an overall decrease in glucose transport into the cells. p38 has been found to regulate the expression of glucose transporter GLUT4 onto the membrane surface for glucose uptake in response to insulin. Hence, this suggests a plausible role in that p38 signalling may be able to explain insulin resistance and the lack of glucose uptake in type II diabetes.^[95]^ Furthermore, another study found the activation of p38 signalling through hepatic neuronal nitric oxide synthase (nNOS) overexpression in HFD obese mice exacerbated glucose tolerance, intrahepatic lipid accumulation through increased triglyceride production, as well as decreased glycogen storage. These phenotypes manifested via a downregulation of hepatic expression of insulin signalling molecules namely p-IRbeta, p-Akt, and p-GSK3beta. This results in hepatic insulin resistance and diabetes, with further metabolic related pathological correlates to hepatic steatosis and non-alcoholic fatty liver disease (NAFLD).^[133]^ However, in another study, when 3T3-L1 adipocytes are isolated from type II diabetic patients, it was observed that there is an increased level of MAPKs signalling as well as p38 activation. This was responsible for the downregulation of GLUT4 expression on the plasma membrane eventually leading to insulin resistance and decreased glucose transport.^[90]^ This observation was consistent with other findings that p38 was elevated in adipocytes of type II diabetic patients.^[52]^ However, in both studies, controversial data highlighted the complexities behind p38-mediated regulation of glucose transport due to insulin stimulation, and as such, p38 and its lack of good *in vivo* models have resulted in a lack of understanding in explaining the molecular mechanism behind the development of insulin resistance.

#### Other MKPs in obesity and diabetes

While several studies have been conducted on MKPs regulation on different MAPKs, as well as their contribution towards the development of both obesity and diabetes, other MKPs studies exist that have yet to establish the type of MAPKs it regulates. However, studies on these MKPs also reveal plausible roles towards the manifestation of obesity and diabetes. In one finding, MKP-3 knockout mice were found to be resistant to diet-induced obesity and displayed improved insulin sensitivity.^[98]^ Conversely, MKP-5 knockout mice develop visceral obesity and insulin resistance with ageing, suggesting its beneficial role in the development of obesity-associated metabolic disorders.^[99]^ MKP-5 has also been shown in obese mice to promote macrophage switching from M1 to M2, thus increasing the expression of anti-inflammatory mediators involved in obesity-induced adipose tissue inflammation, thus potentially reducing obesity, insulin resistance and type II DM.^[101,102]^ In addition, MKP-4 was found to have protective effects against insulin resistance.^[100]^ From these studies, we can observe that different MKPs participate in the development of adipogenesis and insulin resistance distinctly. However, more focused studies need to be conducted to understand the types of MAPKs these MKPs regulate to have a better understanding of the molecular mechanism behind the basis of MKP-mediated regulation towards the contribution of obesity and diabetes.

In summary, the MAPKs pathway plays important roles in regulating adipogenesis and insulin sensitivity under normal physiological processes. Dysregulation of the axis of MAPKs pathways, either through the core components or their regulators, has resulted in altered adipogenesis and adipose tissue homeostasis, which subsequently increased the risk of the development of obesity and diabetes. However, many complexities are interwoven within the pathways that may produce numerous controversial results. To add to the complexities, the development of diabetes is often confounded by other risk factors, which may manifest as a result of a conundrum of signalling pathways. As a result, it is paramount to consider the relative contributions of cross-talks between various pathways for better identification of potential therapeutic targets.

### MAPKs in cardiovascular health

Atherosclerosis is an important pathological process that has been determined to be a major risk factor towards the development of cardiovascular diseases. The development of atherosclerosis involves contributions from many cell types. However, the roles of foam cells in contributing to plaque formation have been identified to be a major contributor, and therefore many studies have focused on this cell type. The roles of MAPKs pathways have been heavily implicated in foam cells development and have been identified via both *in vitro* and *in vivo* studies. Moreover, MAPKs signalling is important for the normal physiological maintenance of the heart. Abnormal activities of MAPK signalling have been reported to contribute importantly towards the development of cardiovascular diseases.^[114]^ Additionally, MAPKs have also been implicated in exacerbating stroke (haemorrhagic and ischaemic) damage.^[114]^

#### ERKs in cardiovascular health

In the milieu of ERK involvement in foam cell maintenance, treatment of mouse macrophages with oxidised low-density lipoprotein (ox-LDL), a method of triggering foam cell formation, resulted in sustained activation of ERK1/2. However, treatment of ERK inhibitor PD98059 has shown limited significant impact on foam cell formation, suggesting that ERK may not be an important contributor to the formation of foam cells.^[114]^

The RAF/MEK/ERK pathway has been reported to be implicated in the development of cardiac hypertrophy. Administration of MEK1/2 inhibitor U0126 or RAF inhibitor SB-386023 resulted in the reduction in cardiac hypertrophy.^[107]^ In addition, Grb2 heterogenous knockout mice also showed resistance to cardiac hypertrophy.^[118]^ However, ERK1 knockout mice and ERK2 heterogeneous knockout mice still displayed cardiac hypertrophy, suggesting that other molecules associated with the pathway, rather than ERK1/2 themselves, may be more important in mediating the cardiac hypertrophic response.^[108]^ On the contrary, overexpression of MEK1 in mice display cardiac hypertrophy,^[109]^ whereas overexpression of MKP-3 which resulted in the reduction in ERK1/2 activation also showed signs of hypertrophy, thus coinciding with the findings that ERK1/2 may not directly participate in cardiac hypertrophy.^[119]^ Besides that, *in vitro* and *in vivo* studies have also illustrated that via the axis of RAS/RAF/MEK/ERK, ion modulators can be altered, resulting in defective sarcoplasmic reticulum calcium homeostasis and this pathological cardiac remodelling provides a basis towards hypertrophic cardiomyopathy, which is often fatal.^[114]^

In addition, ERK5 has not been extensively studied in the development of cardiac hypertrophy. ERK5 activation was detected in aortic smooth muscle cells in cardiac hypertrophic patients. Genetic deletion of ERK5 as well as administration of ERK5 inhibitor BIX02189 display attenuation of cardiac hypertrophy.^[53]^ As discussed above, ERK5 plays an important physiological role in maintaining cardiovascular development as well as vascular integrity. MEK5 constitutive expression in transgenic mice showed increased cardiac hypertrophic responses, however, upon ischaemic/reperfusion injury, constitutive expressed MEK5 transgenic mice inhibited cardiac injury via the activation of ERK5.^[119]^ From these findings, even though the study of ERK5 is still in its infancy stage, it can be observed that ERK5 displays an intricate relationship with heart physiology and development, and much more needs to be done to fully understand its roles. In addition, ERK1/2 have also been found to be elevated following ischaemic stroke. However, the roles of ERK1/2 signalling have been identified to be controversial. ERK1/2 activation can be deemed as neuroprotective by upregulating growth factors or even inducing hypothermia following ischaemia to reduce damage. On the contrary, sustained activation of ERK1/2 has been associated with continuous inflammation and cerebral cell death, and thereby exacerbates the damage induced by ischaemia.^[114]^ Our group has also previously established that inhibition of ERK signalling pathway in neuronal cells subjected to ischaemic conditions resulted in a decrease in the expression of NLRP1 and NLRP3 inflammasomes, which in turn reduces the expression of inflammatory precursors and proteins such as interleukin(IL)-1β and IL-18.^[134]^ As such, given the controversial nature of ERK1/2 in ischaemic stroke, careful consideration needs to be taken to choose ERK1/2 as potential therapeutic targets for ischaemic stroke as toxicity may result due to our lack of understanding of ERK1/2 roles.

#### JNKs in cardiovascular health

The JNK pathways have been implicated in foam cell formation. Like ERK, treatment of mouse macrophages with ox-LDL resulted in sustained activation of JNK1/2 pathways. Treatment of JNK inhibitor SP600125 in different macrophage cell lines block foam cell formation, demonstrating the possible contribution of JNK in the formation of foam cells.^[103]^ Moreover, JNK1/2 knockout mice displayed resistance to atherosclerosis development after being fed a high-cholesterol diet.^[105]^ In JNK2 knockout mice, while the cellular contributions towards lesion formation remain the same, the atherosclerotic lesion found in these animals was smaller.^[135]^ Conversely, the roles of JNK pathways in mediating cardiac hypertrophic responses are much less understood. JNK1/2/3 knockout mice display cardiac hypertrophy response, which is also displayed by wild type mice. Notably, JNK1 knockout mice exhibited lower left ventricular systolic function during pressure overload before returning to basal levels, suggesting that JNK1 may be cardioprotective with respect to cardiac hypertrophy.^[115]^ On the contrary, overexpression of upstream JNK component, MKK7, resulted in congestive heart failure in mice but not hypertrophy.^[109]^ However, this finding is inconsistent with studies on MKK4 showing that cardiomyocytes with JNK overactivation via MKK4 resulted in cardiac hypertrophy, whereas the dominant negative mutant form of MKK4 reduced hypertrophic responses.^[109,116]^ Given that JNK involvement is inconsistent in both *in vitro* and *in vivo* studies, the molecular mechanism of JNK in the development of cardiac hypertrophy is still unclear. However, the roles of JNK in the process of cardiac remodelling is much better understood, where JNK pathway activation in the heart has resulted in cardiomyopathy and extracellular matrix remodelling. JNK1 knockout in the heart of mice resulted in elevated cardiac fibrosis when subjected to pressure overload,^[136]^ whereas treatment of JNK inhibitor SP600125 to dilated cardiomyopathy in hamsters resulted in increased apoptosis and fibrosis.^[111]^ Moreover, in aortic banded rats, treatment of all trans-retinoic acid to rats inhibit MAPK signalling, including the JNK pathway, via the upregulation of MKP-1/2, which in turn prevent the development of cardiac remodelling.^[111]^ Indeed, the roles of MKP-1/2 have been widely associated with cardioprotective functions. MKP-1 knockout mice results in limited cardiac hypertrophic response as compared to their wild type counterpart, whereas MKP-1/2 double knockout mice display immense hypertrophic responses.^[109]^ As such, the roles of MKP-1/2 in the protection against these pathologies seem to be a potential therapeutic target to study. In the area of stroke pathogenesis, our study has previously established that inhibition of p38 signalling pathway in neuronal cells subjected to ischaemic conditions also resulted in a decrease in the expression of NLRP1 and NLRP3 inflammasomes, which in turn reduces the expression of inflammatory precursors and proteins such as IL-1β and IL-18. Therefore, JNK appears to promote inflammatory response and damage in ischaemic conditions.^[134]^

#### p38 in cardiovascular health

Similar to ERK and JNK, treatment of mouse macrophages with ox-LDL resulted in the sustained activation of p38α, and treatment of p38 inhibitor SB203580 in different macrophage cell lines blocked foam cell formation. Therefore, the p38 MAPK pathway is involved in the formation of foam cells as well.^[103]^

As p38α knockout mice display embryonic lethality, *in vivo* studies using MK2, which is a downstream substrate for p38α, also display resistance to atherosclerosis development.^[67]^ However, studies on p38α in macrophages and endothelial cells highlight that p38α is not required in plaque formation.^[137]^ Downregulation of p38α resulted in increased apoptosis of macrophages, suggesting that it plays a pro-survival role in macrophages, which in conjunction with JNK, drives the formation of atherosclerotic lesions.^[104]^ On the contrary, MKP-1-deficient mice in bone marrow transplantation model displayed accelerated atherosclerotic lesion formation after being fed a HFD.^[106]^ Conversely, in an apoE deficient background, MKP-1 deficiency decreases the formation of atherosclerotic lesions.^[138]^ The role of MKP-1 in atherosclerotic lesion formation is therefore unclear. Overall, these studies showed the importance of the MAPKs pathway in atherosclerosis and therefore may be a potential area to explore to attenuate the development of atherosclerosis, and the subsequent risks of heart diseases and even stroke.

Furthermore, p38 seems to be implicated in cardiac damage. p38α/β knockout mice also display similar phenotypes as JNK knockout mice. p38α/β dominant negative overexpressed mice display a cardiac hypertrophic response, which is also displayed by wild type mice.^[118]^ Similarly, overexpression of dominant negative MKK3 and MKK6, upstream activators of p38 pathways also develop cardiac hypertrophy.^[112]^ Similarly, overexpression of p38 in mice does not result in cardiac hypertrophy, which coincides with knockout studies.^[139]^

A study has also found chamber-specific growth associated with selective inactivation of p38 in neonatal mice led to marked increases in cardiomyocyte proliferation and hypertrophy, with eventual adult progression to pulmonary hypertension and right heart failure.^[140]^ Notably, the roles of p38 pathways in inducing cardiac remodelling have also been investigated. p38 has been reported to act via the TGF/TAK1/p38 axis to induce cardiac remodelling.^[141]^ *In vitro* studies have also demonstrated the roles of p38 in inducing pro-inflammatory cytokines production in myocytes via the p38 pathway. Taken together, this may provide the molecular mechanism for the induction of fibrosis and hypertrophy.^[113]^ Indeed, long-term inhibition of p38 using RWJ67657 resulted in attenuation of cardiac remodelling.^[142]^ In another study, treatment of p38 inhibitor SB203580 and FR167653 also displayed reduced cardiac remodelling,^[143]^ all of which suggest that p38 may be a contributor towards the process of pathological cardiac remodelling.

Following a stroke, p38 has been found to be elevated in the peri-infarct area in astrocytes, contributing to astrogliosis, which is a damaging scarring consequence of a stroke. Treatment of p38 inhibitor SB239063 reduced astrogliosis following an ischaemic stroke. Moreover, p38 transgenic mice also display attenuation of astrocyte migration and astrogliosis, which attenuate the overall damage induced by an ischaemic stroke.^[113]^ As such, p38 seems to play a negative role in an ischaemic stroke and is therefore a potential post-stroke therapeutic target. Besides that, it has been identified that MKP-1 plays a protective role in a stroke. Treatment of MKP-1 inhibitor, as well as the genetic ablation of MKP1, worsens stroke outcome by increasing inflammation and apoptosis of neuronal and glial cells through modulation of p38. As such, modulation of MKP-1 levels following an ischaemic stroke may provide a potential approach in reducing ischaemic injury to the brain.^[122]^ As discussed above, inhibition of p38 signalling pathway resulted in a decrease in the expression of NLRP1 and NLRP3 inflammasomes, which in turn reduces the expression of inflammatory precursors and proteins such as IL-1β and IL-18 during ischaemic conditions. Therefore, it is important to also consider the roles of p38 in inducing inflammation and damage during an ischaemic stroke in neuronal cells.^[134]^

### MAPKs in hepatic diseases

NAFLD is a progressive disorder, which belongs to a broad category of liver diseases. Retention of lipids in liver cells termed steatosis, in conjunction with inflammation of the liver because of this hyperlipidaemia state, known as steatohepatitis, are usually present. Chronically, deposition of extracellular matrix and liver scarring known as liver fibrosis will ensue. NAFLD is usually exacerbated by other contributors in metabolic syndrome, such as obesity and diabetes. Progression of this disorder, if left untreated, will lead to numerous complications such as liver cirrhosis, liver failure, and even hepatocarcinoma. NAFLD, as part of the metabolic syndrome, have resulted in alarming morbidity and mortality, and is therefore of great concern.^[144]^

#### JNKs in hepatic diseases

The roles of MAPKs in hepatic diseases are well investigated. JNK has been reported in many studies to be strongly associated with the manifestation of NAFLD. JNK1 has been found to be a contributor towards steatosis development in mice fed with a HFD.^[125]^ In another study, which used methionine- and choline-deficient (MCD) diets in mice to model non-alcoholic steatohepatitis, JNK1 was shown to be responsible for the development of NASH.^[145]^ JNK1 knockdown using antisense oligonucleotide leads to decreased steatohepatitis and insulin resistance even when fed a HFD. Similarly, JNK1 is also reported to be an important mediator of liver injury in MCD mice. However, knockdown of JNK2 displayed steatohepatitis, which had a similar phenotype as wild type mice.^[126]^ Thus, JNK2 does not seem to affect the development of these hepatic diseases, suggesting the differential roles these two JNK isoforms play in the liver. Notably, ablation of JNK2 resulted in increased liver injury through increased apoptosis,^[127]^ thus JNK2 may play an opposing hepatic protective role to JNK1. Indeed, in bone marrow transplant chimeric mice where Kupffer cells were being replaced with JNK1 and JNK2 knockout cells, JNK1 was found to be needed for the progression towards liver fibrosis through induction of chronic inflammation.^[146]^ Putting the data together, JNK1 contributes significantly towards the pathological development of liver diseases and is therefore a potential therapeutic target.

#### p38 in hepatic diseases

p38 has also been reported to be a major contributor towards the pathological development of liver diseases. Knockout experiment of liver-specific p38α in mice resulted in reduced hepatomegaly.^[123]^ p38γ and p38δ knockout mice display hepatosteatosis resistance in HFD as well as MCD diet mice.^[124]^ In both studies, p38 is important in driving chronic inflammation to induce liver damage, with p38γ and p38δ responsible for neutrophil migration to the liver to induce steatosis, as well as p38α upregulating proinflammatory cytokines expression.^[123,124]^ As such, p38 isoforms are also important in the driver of hepatic diseases. Indeed, a recent study reported p38γ and p38δ are selected to be potential therapeutic targets for NAFLD, another study has also reported the use of melatonin in improving NAFLD symptoms in HFD diet mice by modulating p38 pathways. Thus, the therapeutic potential using MAPKs pathway modulation cannot be discounted.

## Future Perspectives

Our metabolic adaptations greatly differ between individuals, and are often confounded by environmental, genetic, and stochastic variables. As a result, disruption in metabolic adaptation may arise, leading to a wide spectrum of metabolic disorders. Many countries have witnessed the seriousness of metabolic disorders plaquing populations, which have resulted in high morbidity and mortality in many people. As focus has been increasingly shifted towards understanding the molecular mechanism(s) behind the pathogenesis of these disorders, the MAPK pathways have been identified as important signalling nodes that have been closely associated with the manifestation of metabolic disorders. While the MAPKs have been studied intensively in the area of oncology and neurology, the investigation of MAPKs in the area of metabolic disorders are sketchy and in their infancy. In addition, results revealed from the study of how MAPK pathways have resulted in the development of metabolic disorders have often yield complexities. Such complexities arise due to the lack of proper animal models to study the roles of MAPKs in disease development, where opposing results of the same targets may be observed in two different models of the same diseases. In addition, the MAPK signalling cascades are important evolutionarily conserved pathways that regulate many important physiological processes within the body, as well as sharing many cross-talks with other signalling pathways. Even though potential therapeutic targets, which are responsible for the development of certain metabolic disorders, can be identified, manipulation of these targets in research studies have often yielded toxicity or compensatory mechanisms due to overlapping functions of a variety of pathways. As such, identification of a single MAPK signalling that accounts for the development of metabolic disorders may be difficult. However, recent studies have shifted the attention towards understanding the regulations governing the activities of MAPKs. It was discovered that aberration in these regulatory molecules have disrupted the normal signalling axis of MAPKs, and as a result, contributes to the development of various metabolic disorders. In addition, as these regulatory mechanisms often confer specificity in certain MAPKs, manipulation of these regulatory targets have shown promising results by reducing the probability of involving other cross-talk pathways. Therefore, it is paramount that future studies need to also consider the regulatory mechanisms behind such pathways. While the investigation of potential therapeutic targets within the MAPK signalling axis have been initiated recently amongst the metabolic disorders’ milieu, the silver lining exists in that many promising targets are being identified. The future challenges lie in understanding the complexities that govern the function of these potential therapeutic elements, as well as how such elements are interconnected and regulated. Hopefully, therapeutic intervention can be quickly discovered for the attenuation of these metabolic disorders and reduce the social and economic burden in many countries.

## References

[1] Cargnello M, Roux PP (2011). Activation and function of the MAPKs and their substrates, the MAPK-activated protein kinases. Microbiol. Mol. Biol. Rev.

[2] Turjanski AG, Vaqué JP, Gutkind JS (2007). MAP kinases and the control of nuclear events. Oncogene.

[3] Kasler HG, Victoria J, Duramad O, Winoto A (2000). ERK5 is a novel type of mitogen-activated protein kinase containing a transcriptional activation domain. Mol. Cell. Biol.

[4] Santos SDM, Verveer PJ, Bastiaens PIH (2007). Growth factor-induced MAPK network topology shapes Erk response determining PC-12 cell fate. Nat. Cell Biol.

[5] Saud K, Herrera-Molina R, Von Bernhardi R (2005). Pro- and anti-inflammatory cytokines regulate the ERK pathway: implication of the timing for the activation of microglial cells. Neurotox. Res.

[6] Son Y, Cheong YK, Kim NH, Chung HT, Kang DG, Pae HO (2011). Mitogen-activated protein kinases and reactive oxygen species: how can ROS activate MAPK pathways?. J. Signal Transduct.

[7] Han MY, Kosako H, Watanabe T, Hattori S (2007). Extracellular signal-regulated kinase/mitogen-activated protein kinase regulates actin organization and cell motility by phosphorylating the actin cross-linking protein EPLIN. Mol. Cell. Biol.

[8] Caunt CJ, Finch AR, Sedgley KR, McArdle CA (2006). Seven-transmembrane receptor signalling and ERK compartmentalization. Trends Endocrinol. Metab.

[9] Sabio G, Davis RJ (2014). TNF and MAP kinase signalling pathways. Semin. Immunol.

[10] Margolis B, Skolnik EY (1994). Activation of Ras by receptor tyrosine kinases. J. Am. Soc. Nephrol.

[11] Baljuls A, Kholodenko BN, Kolch W (2013). It takes two to tango--signalling by dimeric Raf kinases. Mol. Biosyst.

[12] Butch ER, Guan KL (1996). Characterization of ERK1 activation site mutants and the effect on recognition by MEK1 and MEK2. J. Biol. Chem.

[13] Roskoski R (2012). MEK1/2 dual-specificity protein kinases: structure and regulation. Biochem. Biophys. Res. Commun.

[14] Golding SE, Morgan RN, Adams BR, Hawkins AJ, Povirk LF, Valerie K (2009). Pro-survival AKT and ERK signaling from EGFR and mutant EGFRvIII enhances DNA double-strand break repair in human glioma cells. Cancer Biol. Ther.

[15] Hawkins AJ, Golding SE, Khalil A, Valerie K (2011). DNA double-strand break - induced pro-survival signaling. Radiother. Oncol.

[16] Ewings KE, Wiggins CM, Cook SJ (2007). Bim and the pro-survival Bcl-2 proteins: opposites attract, ERK repels. Cell Cycle.

[17] Jin R, Liu L, Zhang S, Nanda A, Li G (2013). Role of inflammation and its mediators in acute ischemic stroke. J. Cardiovasc. Transl. Res.

[18] Cagnol S, Chambard JC (2010). ERK and cell death: mechanisms of ERK-induced cell death--apoptosis, autophagy and senescence. FEBS J.

[19] Monje P, Hernández-Losa J, Lyons RJ, Castellone MD, Gutkind JS (2005). Regulation of the transcriptional activity of c-Fos by ERK. A novel role for the prolyl isomerase PIN1. J. Biol. Chem.

[20] Brami-Cherrier K, Roze E, Girault JA, Betuing S, Caboche J (2009). Role of the ERK/MSK1 signalling pathway in chromatin remodelling and brain responses to drugs of abuse. J. Neurochem.

[21] Fischer AM, Katayama CD, Pagès G, Pouysségur J, Hedrick SM (2005). The role of erk1 and erk2 in multiple stages of T cell development. Immunity.

[22] Nekrasova T, Shive C, Gao Y, Kawamura K, Guardia R, Landreth G (2005). ERK1-deficient mice show normal T cell effector function and are highly susceptible to experimental autoimmune encephalomyelitis. J. Immunol.

[23] Mazzucchelli C, Vantaggiato C, Ciamei A, Fasano S, Pakhotin P, Krezel W (2002). Knockout of ERK1 MAP kinase enhances synaptic plasticity in the striatum and facilitates striatal-mediated learning and memory. Neuron.

[24] Vithayathil J, Pucilowska J, Goodnough LH, Atit RP, Landreth GE (2015). Dentate gyrus development requires ERK activity to maintain progenitor population and MAPK pathway feedback regulation. J. Neurosci.

[25] Bost F, Aouadi M, Caron L, Binétruy B (2005). The role of MAPKs in adipocyte differentiation and obesity. Biochimie.

[26] Saba-El-Leil MK, Vella FD, Vernay B, Voisin L, Chen L, Labrecque N (2003). An essential function of the mitogen-activated protein kinase Erk2 in mouse trophoblast development. EMBO Rep.

[27] Hatano N, Mori Y, Oh-hora M, Kosugi A, Fujikawa T, Nakai N (2003). Essential role for ERK2 mitogen-activated protein kinase in placental development. Genes Cells.

[28] Satoh A, Brace CS, Rensing N, Cliften P, Wozniak DF, Herzog ED (2013). Sirt1 extends life span and delays aging in mice through the regulation of Nk2 homeobox 1 in the DMH and LH. Cell Metab.

[29] Dong C, Yang DD, Tournier C, Whitmarsh AJ, Xu J, Davis RJ (2000). JNK is required for effector T-cell function but not for T-cell activation. Nature.

[30] Sadoshima J, Montagne O, Wang Q, Yang G, Warden J, Liu J (2002). The MEKK1-JNK pathway plays a protective role in pressure overload but does not mediate cardiac hypertrophy. J. Clin. Invest.

[31] Coffey ET (2014). Nuclear and cytosolic JNK signalling in neurons. Nat. Rev. Neurosci.

[32] Solinas G, Becattini B (2017). JNK at the crossroad of obesity, insulin resistance, and cell stress response. Mol. Metab.

[33] Chromik AM, Müller AM, Körner J, Belyaev O, Holland-Letz T, Schmitz F (2007). Genetic deletion of JNK1 and JNK2 aggravates the DSS-induced colitis in mice. J. Invest. Surg.

[34] Guma M, Ronacher LM, Firestein GS, Karin M, Corr M (2011). JNK-1 deficiency limits macrophage-mediated antigen-induced arthritis. Arthritis Rheum.

[35] Sancho R, Nateri AS, de Vinuesa AG, Aguilera C, Nye E, Spencer-Dene B (2009). JNK signalling modulates intestinal homeostasis and tumourigenesis in mice. EMBO J.

[36] Jaeschke A, Rincón M, Doran B, Reilly J, Neuberg D, Greiner DL (2005). Disruption of the Jnk2 (Mapk9) gene reduces destructive insulitis and diabetes in a mouse model of type I diabetes. Proc. Natl. Acad. Sci. U. S. A.

[37] Ismail HM, Zarebska J, Vincent T, Saklatvala J (2015). JNK2 knockout mice are significantly protected from surgically induced osteoarthritis. Osteoarthritis Cartilage.

[38] Peng J, Andersen JK (2003). The role of c-Jun N-terminal kinase (JNK) in Parkinson’s disease. IUBMB Life.

[39] Sherrin T, Blank T, Hippel C, Rayner M, Davis RJ, Todorovic C (2010). Hippocampal c-Jun-N-terminal kinases serve as negative regulators of associative learning. J. Neurosci.

[40] Genabai NK, Ahmad S, Zhang Z, Jiang X, Gabaldon CA, Gangwani L (2015). Genetic inhibition of JNK3 ameliorates spinal muscular atrophy. Hum. Mol. Genet.

[41] Yang DD, Kuan CY, Whitmarsh AJ, Rincón M, Zheng TS, Davis RJ (1997). Absence of excitotoxicity-induced apoptosis in the hippocampus of mice lacking the Jnk3 gene. Nature.

[42] Kowalski J, McBryan T, Nelson DM, Vanderkraats ND, Shah PP, van Tuyn J (2013). Senescent cells harbour features of the cancer epigenome. Nat. Cell Biol.

[43] Uddin S, Ah-Kang J, Ulaszek J, Mahmud D, Wickrema A (2004). Differentiation stage-specific activation of p38 mitogen-activated protein kinase isoforms in primary human erythroid cells. Proc. Natl. Acad. Sci. U. S. A.

[44] Mudgett JS, Ding J, Guh-Siesel L, Chartrain NA, Yang L, Gopal S (2000). Essential role for p38alpha mitogen-activated protein kinase in placental angiogenesis. Proc. Natl. Acad. Sci.

[45] Haines JD, Fragoso G, Hossain S, Mushynski WE, Almazan G (2008). p38 Mitogen-activated protein kinase regulates myelination. J. Mol. Neurosci.

[46] Gupta J, del Barco Barrantes I, Igea A, Sakellariou S, Pateras IS, Gorgoulis VG (2014). Dual function of p38α MAPK in colon cancer: suppression of colitis-associated tumor initiation but requirement for cancer cell survival. Cancer Cell.

[47] Roche O, Fernández-Aroca DM, Arconada-Luque E, García-Flores N, Mellor LF, Ruiz-Hidalgo MJ (2020). p38β and cancer: the beginning of the road. Int. J. Mol. Sci.

[48] Perdiguero E, Ruiz-Bonilla V, Gresh L, Hui L, Ballestar E, Sousa-Victor P (2007). Genetic analysis of p38 MAP kinases in myogenesis: fundamental role of p38alpha in abrogating myoblast proliferation. EMBO J.

[49] Pogozelski AR, Geng T, Li P, Yin X, Lira VA, Zhang M (2009). P38gamma mitogen-activated protein kinase is a key regulator in skeletal muscle metabolic adaptation in mice. PLoS One.

[50] Schindler EM, Hindes A, Gribben EL, Burns CJ, Yin Y, Lin MH (2009). P38delta mitogen-activated protein kinase is essential for skin tumor development in mice. Cancer Res.

[51] Sohn SJ, Sarvis BK, Cado D, Winoto A (2002). ERK5 MAPK regulates embryonic angiogenesis and acts as a hypoxia-sensitive repressor of vascular endothelial growth factor expression. J. Biol. Chem.

[52] Fröjdö S, Vidal H, Pirola L (2009). Alterations of insulin signaling in type 2 diabetes: a review of the current evidence from humans. Biochim. Biophys. Acta.

[53] Li T, Pan YW, Wang W, Abel G, Zou J, Xu L (2013). Targeted deletion of the ERK5 MAP kinase impairs neuronal differentiation, migration, and survival during adult neurogenesis in the olfactory bulb. PLoS One.

[54] Lin ECK, Amantea CM, Nomanbhoy TK, Weissig H, Ishiyama J, Hu Y (2016). ERK5 kinase activity is dispensable for cellular immune response and proliferation. Proc. Natl. Acad. Sci. U. S. A.

[55] Bost F, Aouadi M, Caron L, Even P, Belmonte N, Prot M (2005). The extracellular signal-regulated kinase isoform ERK1 is specifically required for in vitro and in vivo adipogenesis. Diabetes.

[56] Satoh Y, Endo S, Ikeda T, Yamada K, Ito M, Kuroki M (2007). Extracellular signal-regulated kinase 2 (ERK2) knockdown mice show deficits in long-term memory; ERK2 has a specific function in learning and memory. J. Neurosci.

[57] Hisamoto N, Nagamori Y, Shimizu T, Pastuhov SI, Matsumoto K (2016). The C. elegans discoidin domain receptor DDR-2 modulates the met-like RTK-JNK signaling pathway in axon regeneration. PLoS Genet.

[58] Gutkind JS (1998). The pathways connecting G protein-coupled receptors to the nucleus through divergent mitogen-activated protein kinase cascades. J. Biol. Chem.

[59] Tournier C, Dong C, Turner TK, Jones SN, Flavell RA, Davis RJ (2001). MKK7 is an essential component of the JNK signal transduction pathway activated by proinflammatory cytokines. Genes Dev.

[60] Zhu J, Zhang J, Huang H, Li J, Yu Y, Jin H (2014). Crucial role of c-Jun phosphorylation at Ser63/73 mediated by PHLPP protein degradation in the cheliensisin a inhibition of cell transformation. Cancer Prev. Res. (Phila).

[61] Thalhamer T, McGrath MA, Harnett MM (2008). MAPKs and their relevance to arthritis and inflammation. Rheumatology (Oxford).

[62] Han MS, Jung DY, Morel C, Lakhani SA, Kim JK, Flavell RA (2013). JNK expression by macrophages promotes obesity-induced insulin resistance and inflammation. Science.

[63] Quigley HA, Cone FE, Gelman SE, Yang Z, Son JL, Oglesby EN (2011). Lack of neuroprotection against experimental glaucoma in c-Jun N-terminal kinase 3 knockout mice. Exp. Eye Res.

[64] Liu J, Lin A (2005). Role of JNK activation in apoptosis: a double-edged sword. Cell Res.

[65] Cuenda A, Rousseau S (2007). p38 MAP-Kinases pathway regulation, function and role in human diseases. Biochim. Biophys. Acta.

[66] Zarubin T, Han J (2005). Activation and signaling of the p38 MAP kinase pathway. Cell Res.

[67] Gupta J, Nebreda AR (2015). Roles of p38$α$ mitogen-activated protein kinase in mouse models of inflammatory diseases and cancer. FEBS J.

[68] Nishimoto S, Nishida E (2006). MAPK signalling: ERK5 versus ERK1/2. EMBO Rep.

[69] Hayashi M, Kim SW, Imanaka-Yoshida K, Yoshida T, Abel ED, Eliceiri B (2004). Targeted deletion of BMK1/ERK5 in adult mice perturbs vascular integrity and leads to endothelial failure. J. Clin. Invest.

[70] Katz-jaffe MG, Linck DW, Schoolcraft WB, Gardner DK (2004). Embryonic development. Reproduction.

[71] Nishimoto S, Kusakabe M, Nishida E (2005). Requirement of the MEK5–ERK5 pathway for neural differentiation in Xenopus embryonic development. EMBO Rep.

[72] Coulombe P, Meloche S (2007). Atypical mitogen-activated protein kinases: structure, regulation and functions. Biochim. Biophys. Acta.

[73] Brown MD, Sacks DB (2009). Protein scaffolds in MAP kinase signalling. Cell. Signal.

[74] Wu C, Lai CF, Mobley WC (2001). Nerve growth factor activates persistent Rap1 signaling in endosomes. J. Neurosci.

[75] Hunter T (1995). Protein kinases and phosphatases: the Yin and Yang of protein phosphorylation and signaling. Cell.

[76] Patterson KI, Brummer T, O’Brien PM, Daly RJ (2009). Dual-specificity phosphatases: critical regulators with diverse cellular targets. Biochem. J.

[77] Low HB, Zhang Y (2016). Regulatory roles of MAPK phosphatases in cancer. Immune Netw.

[78] Li CY, Yang LC, Guo K, Wang YP, Li YG (2015). Mitogen-activated protein kinase phosphatase-1: a critical phosphatase manipulating mitogen-activated protein kinase signaling in cardiovascular disease (Review). Int. J. Mol. Med.

[79] Kamata H, Honda S, Maeda S, Chang L, Hirata H, Karin M (2005). Reactive oxygen species promote TNFalpha-induced death and sustained JNK activation by inhibiting MAP kinase phosphatases. Cell.

[80] Udenwobele DI, Su RC, Good SV, Ball TB, Varma Shrivastav S, Shrivastav A (2017). Myristoylation: an important protein modification in the immune response. Front. Immunol.

[81] Fantz DA, Jacobs D, Glossip D, Kornfeld K (2001). Docking sites on substrate proteins direct extracellular signal-regulated kinase to phosphorylate specific residues. J. Biol. Chem.

[82] Whitmarsh AJ (2007). Regulation of gene transcription by mitogen-activated protein kinase signaling pathways. Biochim. Biophys. Acta.

[83] Sharrocks SH, Sharrocks AD (2004). SUMO promotes HDAC-mediated transcriptional repression. Mol. Cell.

[84] Yang SH, Sharrocks AD (2005). PIASx acts as an Elk-1 coactivator by facilitating derepression. EMBO J.

[85] Cornier MA, Dabelea D, Hernandez TL, Lindstrom RC, Steig AJ, Stob NR (2008). The metabolic syndrome. Endocr. Rev.

[86] Gehart H, Kumpf S, Ittner A, Ricci R (2010). MAPK signalling in cellular metabolism: stress or wellness?. EMBO Rep.

[87] Prusty D, Park BH, Davis KE, Farmer SR (2002). Activation of MEK/ERK signaling promotes adipogenesis by enhancing peroxisome proliferator-activated receptor ?? (PPAR??) and C/EBP?? gene expression during the differentiation of 3T3-L1 preadipocytes. J. Biol. Chem.

[88] Zhu H, Guariglia S, Li W, Brancho D, Wang ZV, Scherer PE (2014). Role of extracellular signal-regulated kinase 5 in adipocyte signaling. J. Biol. Chem.

[89] Engelman JA, Berg AH, Lewis RY, Lin A, Lisanti MP, Scherer PE (1999). Constitutively active mitogen-activated protein kinase kinase 6 (MKK6) or salicylate induces spontaneous 3T3-L1 adipogenesis. J. Biol. Chem.

[90] Carlson CJ, Koterski S, Sciotti RJ, Poccard GB, Rondinone CM (2003). Enhanced basal activation of mitogen-activated protein kinases in adipocytes from type 2 diabetes: potential role of p38 in the downregulation of GLUT4 expression. Diabetes.

[91] Roth Flach RJ, Bennett AM (2010). Mitogen-activated protein kinase phosphatase-1 – a potential therapeutic target in metabolic disease. Expert Opin. Ther. Targets.

[92] Jaeschke A, Czech MP, Davis RJ (2004). An essential role of the JIP1 scaffold protein for JNK activation in adipose tissue. Genes Dev.

[93] Wu JJ, Roth RJ, Anderson EJ, Hong EG, Lee MK, Choi CS (2006). Mice lacking MAP kinase phosphatase-1 have enhanced MAP kinase activity and resistance to diet-induced obesity. Cell Metab.

[94] Gold MR (2008). B cell development: important work for ERK. Immunity.

[95] Niu W, Huang C, Nawaz Z, Levy M, Somwar R, Li D (2003). Maturation of the regulation of GLUT4 activity by p38 MAPK during L6 cell myogenesis. J. Biol. Chem.

[96] Kaneto H, Matsuoka TA, Nakatani Y, Kawamori D, Matsuhisa M, Yamasaki Y (2005). Oxidative stress and the JNK pathway in diabetes. Curr. Diabetes Rev.

[97] Rodriguez A, Durán A, Selloum M, Champy MF, Diez-Guerra FJ, Flores JM (2006). Mature-onset obesity and insulin resistance in mice deficient in the signaling adapter p62. Cell Metab.

[98] Feng B, Jiao P, Helou Y, Li Y, He Q, Walters MS (2014). Mitogen-activated protein kinase phosphatase 3 (MKP-3)-deficient mice are resistant to diet-induced obesity. Diabetes.

[99] Zhang Y, Nguyen T, Tang P, Kennedy NJ, Jiao H, Zhang M (2015). Regulation of adipose tissue inflammation and insulin resistance by MAPK phosphatase 5. J. Biol. Chem.

[100] Emanuelli B, Eberlé D, Suzuki R, Kahn CR (2008). Overexpression of the dual-specificity phosphatase MKP-4/DUSP-9 protects against stress-induced insulin resistance. Proc. Natl. Acad. Sci. U. S. A.

[101] Zatterale F, Longo M, Naderi J, Raciti GA, Desiderio A, Miele C (2019). Chronic adipose tissue inflammation linking obesity to insulin resistance and type 2 diabetes. Front. Physiol.

[102] Lu Y, Ma J, Zhao J, Song Z, Zhou C, Liu X (2020). The role of MKP-5 in adipocyte-macrophage interactions during obesity. Obes. Facts.

[103] Zhao M, Liu Y, Wang X, New L, Han J, Brunk UT (2002). Activation of the p38 MAP kinase pathway is required for foam cell formation from macrophages exposed to oxidized LDL. APMIS.

[104] Seimon TA, Wang Y, Han S, Senokuchi T, Schrijvers DM, Kuriakose G (2009). Macrophage deficiency of p38alpha MAPK promotes apoptosis and plaque necrosis in advanced atherosclerotic lesions in mice. J. Clin. Invest.

[105] Kwok KHM, Cheng KKY, Hoo RLC, Ye D, Xu A, Lam KSL (2016). Adipose-specific inactivation of JNK alleviates atherosclerosis in apoE-deficient mice. Clin. Sci.

[106] Kim HS, Tavakoli S, Piefer LA, Nguyen HN, Asmis R (2016). Monocytic MKP-1 is a sensor of the metabolic environment and regulates function and phenotypic fate of monocyte-derived macrophages in atherosclerosis. Sci. Rep.

[107] Wang Y (2007). Mitogen-activated protein kinases in heart development and diseases. Circulation.

[108] Mutlak M, Kehat I (2015). Extracellular signal-regulated kinases 1/2 as regulators of cardiac hypertrophy. Front. Pharmacol.

[109] Rose BA, Force T, Wang Y (2010). Mitogen-activated protein kinase signaling in the heart: angels versus demons in a heart-breaking tale. Physiol. Rev.

[110] Nicol RL, Frey N, Pearson G, Cobb M, Richardson J, Olson EN (2001). Activated MEK5 induces serial assembly of sarcomeres and eccentric cardiac hypertrophy. EMBO J.

[111] Kyoi S, Otani H, Matsuhisa S, Akita Y, Tatsumi K, Enoki C (2006). Opposing effect of p38 MAP kinase and JNK inhibitors on the development of heart failure in the cardiomyopathic hamster. Cardiovasc. Res.

[112] Braz JC, Bueno OF, Liang Q, Wilkins BJ, Dai YS, Parsons S (2003). Targeted inhibition of p38 MAPK promotes hypertrophic cardiomyopathy through upregulation of calcineurin-NFAT signaling. J. Clin. Invest.

[113] Li M, Georgakopoulos D, Lu G, Hester L, Kass DA, Hasday J (2005). p38 MAP kinase mediates inflammatory cytokine induction in cardiomyocytes and extracellular matrix remodeling in heart. Circulation.

[114] Sawe N, Steinberg G, Zhao H (2008). Dual roles of the MAPK/ERK1/2 cell signaling pathway after stroke. J. Neurosci. Res.

[115] Liang Q, Bueno OF, Wilkins BJ, Kuan CY, Xia Y, Molkentin JD (2003). c-Jun N-terminal kinases (JNK) antagonize cardiac growth through cross-talk with calcineurin-NFAT signaling. EMBO J.

[116] Liu W, Zi M, Jin J, Prehar S, Oceandy D, Kimura TE (2009). Cardiac-specific deletion of mkk4 reveals its role in pathological hypertrophic remodeling but not in physiological cardiac growth. Circ. Res.

[117] Choudhary R, Palm-Leis A, Scott RC, Guleria RS, Rachut E, Baker KM (2008). All-trans retinoic acid prevents development of cardiac remodeling in aortic banded rats by inhibiting the renin-angiotensin system. Am. J. Physiol. Heart. Circ. Physiol.

[118] Zhang S, Weinheimer C, Courtois M, Kovacs A, Zhang CE, Cheng AM (2003). The role of the Grb2 – p38 MAPK signaling pathway in cardiac hypertrophy and fibrosis. J. Clin. Invest.

[119] Maillet M, Purcell NH, Sargent MA, York AJ, Bueno OF, Molkentin JD (2008). DUSP6 (MKP3) null mice show enhanced ERK1/2 phosphorylation at baseline and increased myocyte proliferation in the heart affecting disease susceptibility. J. Biol. Chem.

[120] Fann DY, Ng GY, Poh L, Arumugam TV (2017). Positive effects of intermittent fasting in ischemic stroke. Exp. Gerontol.

[121] Pezzini A, Grassi M, Del Zotto E, Giossi A, Monastero R, Dalla Volta G (2007). Migraine mediates the influence of C677T MTHFR genotypes on ischemic stroke risk with a stroke-subtype effect. Stroke.

[122] Liu L, Doran S, Xu Y, Manwani B, Ritzel R, Benashski S (2014). Inhibition of mitogen-activated protein kinase phosphatase-1 (MKP-1) increases experimental stroke injury. Exp. Neurol.

[123] Tormos AM, Arduini A, Talens-Visconti R, del Barco Barrantes I, Nebreda AR, Sastre J (2013). Liver-specific p38α deficiency causes reduced cell growth and cytokinesis failure during chronic biliary cirrhosis in mice. Hepatology.

[124] González-Terán B, Matesanz N, Nikolic I, Verdugo MA, Sreeramkumar V, Hernández-Cosido L (2016). p38γ and p38δ reprogram liver metabolism by modulating neutrophil infiltration. EMBO J.

[125] Sun H, Wang X, Chen J, Song K, Gusdon AM, Li L (2016). Melatonin improves non-alcoholic fatty liver disease via MAPK-JNK/P38 signaling in high-fat-diet-induced obese mice. Lipids Health Dis.

[126] Schattenberg JM, Singh R, Wang Y, Lefkowitch JH, Rigoli RM, Scherer PE (2006). JNK1 but not JNK2 promotes the development of steatohepatitis in mice. Hepatology.

[127] Liedtke C, Trautwein C (2006). The role of JNK2 in toxic liver injury. J. Hepatol.

[128] Kodama Y, Brenner DA (2009). c-Jun N-terminal kinase signaling in the pathogenesis of nonalcoholic fatty liver disease: multiple roles in multiple steps. Hepatology.

[129] Qatanani M, Lazar MA (2007). Mechanisms of obesity-associated insulin resistance: many choices on the menu. Genes Dev.

[130] Frémin C, Saba-El-Leil MK, Lévesque K, Ang SL, Meloche S (2015). Functional redundancy of ERK1 and ERK2 MAP kinases during development. Cell Rep.

[131] Horie T, Park G, Inaba Y, Hashiuchi E, Iezaki T, Tokumura K (2019). MAPK Erk5 in leptin receptor-expressing neurons controls body weight and systemic energy homeostasis in female mice. Endocrinology.

[132] Maekawa T, Jin W, Ishii S (2010). The role of ATF-2 family transcription factors in adipocyte differentiation: antiobesity effects of p38 inhibitors. Mol. Cell. Biol.

[133] Zhao T, Li Q, Mao Q, Mu K, Wang C (2021). Hepatic nNOS impaired hepatic insulin sensitivity through the activation of p38 MAPK. J. Endocrinol.

[134] Fann DYW, Lim YA, Cheng YL, Lok KZ, Chunduri P, Baik SH (2018). Evidence that NF-κB and MAPK signaling promotes NLRP inflammasome activation in neurons following ischemic stroke. Mol. Neurobiol.

[135] Babaev VR, Yeung M, Erbay E, Ding L, Zhang Y, May JM (2016). Jnk1 deficiency in hematopoietic cells suppresses macrophage apoptosis and increases atherosclerosis in low-density lipoprotein receptor null mice. Arterioscler. Thromb. Vasc. Biol.

[136] Liu X, Zhang CS, Lu C, Lin SC, Wu JW, Wang ZX (2016). A conserved motif in JNK/p38-specific MAPK phosphatases as a determinant for JNK1 recognition and inactivation. Nat. Commun.

[137] Kardakaris R, Gareus R, Xanthoulea S, Pasparakis M (2011). Endothelial and macrophage-specific deficiency of P38α MAPK does not affect the pathogenesis of atherosclerosis in ApoE−/− mice. PLoS One.

[138] Shen J, Chandrasekharan UM, Ashraf MZ, Long E, Morton RE, Liu Y (2010). Lack of mitogen-activated protein kinase phosphatase-1 protects ApoE-null mice against atherosclerosis. Circ. Res.

[139] Arabacilar P, Marber M (2015). The case for inhibiting p38 mitogen-activated protein kinase in heart failure. Front. Pharmacol.

[140] Yokota T, Li J, Huang J, Xiong Z, Zhang Q, Chan T (2020). p38 Mitogen-activated protein kinase regulates chamber-specific perinatal growth in heart. J. Clin. Invest.

[141] Matsumoto-Ida M, Takimoto Y, Aoyama T, Akao M, Takeda T, Kita T (2006). Activation of TGF-beta1-TAK1-p38 MAPK pathway in spared cardiomyocytes is involved in left ventricular remodeling after myocardial infarction in rats. Am. J. Physiol. Heart Circ. Physiol.

[142] Kompa AR, See F, Lewis DA, Adrahtas A, Cantwell DM, Wang BH (2008). Long-term but not short-term p38 mitogen-activated protein kinase inhibition improves cardiac function and reduces cardiac remodeling post-myocardial infarction. J. Pharmacol. Exp. Ther.

[143] Lu J, Shimpo H, Shimamoto A, Chong AJ, Hampton CR, Spring DJ (2004). Specific inhibition of p38 mitogen-activated protein kinase with FR167653 attenuates vascular proliferation in monocrotaline-induced pulmonary hypertension in rats. J. Thorac. Cardiovasc. Surg.

[144] Zeng L, Tang WJ, Yin JJ, Zhou BJ (2014). Signal transductions and nonalcoholic fatty liver: a mini-review. Int. J. Clin. Exp. Med.

[145] Ma TC, Buescher JL, Oatis B, Funk JA, Nash AJ, Carrier RL (2007). Metformin therapy in a transgenic mouse model of Huntington’s disease. Neurosci. Lett.

[146] Brenner DA, Seki E, Taura K, Kisseleva T, Deminicis S, Iwaisako K (2011). Non-alcoholic steatohepatitis-induced fibrosis: toll-like receptors, reactive oxygen species and Jun N-terminal kinase. Hepatol. Res.

